# Metabolic Profiling of Water-Soluble Compounds from the Extracts of Dark Septate Endophytic Fungi (DSE) Isolated from Scots Pine (*Pinus sylvestris* L.) Seedlings Using UPLC–Orbitrap–MS

**DOI:** 10.3390/molecules24122330

**Published:** 2019-06-25

**Authors:** Jenni Tienaho, Maarit Karonen, Riina Muilu–Mäkelä, Kristiina Wähälä, Eduardo Leon Denegri, Robert Franzén, Matti Karp, Ville Santala, Tytti Sarjala

**Affiliations:** 1 Faculty of Natural Sciences and Engineering, Tampere University, FI-33101 Tampere, Finland; karpmatti1@gmail.com (M.K.); ville.santala@tuni.fi (V.S.); 2 Natural Resources Institute Finland (Luke), FI-00791 Helsinki, Finland; riina.muilu-makela@luke.fi (R.M.-M.); tytti.sarjala@luke.fi (T.S.); 3 Natural Chemistry Research Group, Department of Chemistry, University of Turku, FI-20014 Turku, Finland; maarit.karonen@utu.fi; 4 Department of Chemistry, University of Helsinki, FI-00014 Helsinki, Finland; kristiina.wahala@helsinki.fi (K.W.); eleondenegri@outlook.com (E.L.D.); 5 School of Chemical Engineering, Department of Chemistry and Materials Science, Aalto University, FI-00076 Espoo, Finland; robert.franzen@aalto.fi

**Keywords:** endophytes, endophytic fungi, *Acephala applanata*, *Phialocephala fortinii*, *Humicolopsis cephalosporioides*, *Coniochaeta mutabilis*, Scots pine, metabolites, UPLC–MS, peptides

## Abstract

Endophytes are microorganisms living inside plant hosts and are known to be beneficial for the host plant vitality. In this study, we isolated three endophytic fungus species from the roots of Scots pine seedlings growing on Finnish drained peatland setting. The isolated fungi belonged to dark septate endophytes (DSE). The metabolic profiles of the hot water extracts of the fungi were investigated using Ultrahigh Performance Liquid Chromatography with Diode Array Detection and Electron Spray Ionization source Mass Spectrometry with Orbitrap analyzer (UPLC–DAD–ESI–MS–Orbitrap). Out of 318 metabolites, we were able to identify 220, of which a majority was amino acids and peptides. Additionally, opine amino acids, amino acid quinones, Amadori compounds, cholines, nucleobases, nucleosides, nucleotides, siderophores, sugars, sugar alcohols and disaccharides were found, as well as other previously reported metabolites from plants or endophytes. Some differences of the metabolic profiles, regarding the amount and identity of the found metabolites, were observed even though the fungi were isolated from the same host. Many of the discovered metabolites have been described possessing biological activities and properties, which may make a favorable contribution to the host plant nutrient availability or abiotic and biotic stress tolerance.

## 1. Introduction

Endophytes are bacterial or fungal microorganisms that colonize a wide variety of plant tissues during at least some period of their lifecycle. Endophytic infection is considered inconspicuous, the infected host tissues are at least transiently symptomless, and the microbial colonization is internal [1,2]. Endophytes have been isolated from all of the studied plant species [3]. However, it has been estimated that only 1–2% of the known 300,000 plant species have been studied for their endophytes [4]. The relationship between the host and the endophyte can have many forms ranging from saprobic to parasitic and from exploitative to mutualistic. Endophytic fungi and bacteria have been shown to improve the health of the host plant by improving the biotic and abiotic stress tolerance due to phytohormone production, and host’s nutrient uptake [5,6,7,8]. The endophytes have also been shown to produce toxic chemicals preventing attacks by insects and herbivores [9,10].

Dark septate endophytic fungi (DSE) are often dominant in the roots of tree species [11] and characterized with melanized and septate hyphae. These Ascomycetous conidial or sterile endophytic fungi colonize the roots of many higher plant species widely in the northern hemisphere and are extensively distributed in coniferous boreal forests [12]. The most frequent DSE in natural forest ecosystems in the northern hemisphere belong to the *Phialocephala fortinii* s.l.–*Acephala applanata* species complex (PAC) and up to 80% of fine roots in forest stands can be colonized by them [11]. Studies have shown that DSE and PAC species induce resistance to abiotic stress, accelerate root turnover and mineralization, and suppress root pathogens [11,13,14,15].

The metabolic profiling of endophytes has revealed novel compounds possessing interesting bioactive properties to be utilized in future. For example, during the years 1995 to 2011, at least 313 novel compounds were isolated and identified from bacterial and fungal endophytic microorganisms [16]. These were found possessing interesting properties, for example as agrochemicals, antiparasitics and in the field of pharmacology. Additionally, in the previous studies of Scots pine bacterial endophytes, they have been shown to produce efficient antioxidant and antimicrobial compounds [17]. Water extraction has been previously found effective in yielding bioactive principles from microorganisms. For example, water-soluble nucleosides, exopolysaccharides, peptides, proteins and sterols showed also bioactive properties in the caterpillar parasitic fungus *Cordyceps sinensis* [18].

In this study, we explored three common endophytic fungal isolates from conifer roots and their aqueous extracts using LC–MS methodologies. The endophytic fungal species used in this study were isolated from the roots of eight-year-old Scots pine seedlings growing in a Finnish drained peatland setting. The growth conditions for trees and especially young seedlings in drained peatlands are harsh due to the extreme variability in temperature, solar radiation, variability in soil ground water level (drought/flood) and poor nutrition. The northern peatlands are rather unexplored environments as regards their endophytes. Scots pine is one of the most economically important and common tree species in Finland and the boreal zone in general. In spite of that, a limited number of studies has been published about its endophytic symbionts. In fact, to our knowledge, this is the first time that the metabolic profile of water extracted Scots pine root associated endophytic fungi belonging to DSE is investigated. However, under this kind of continuously strenuous growth conditions, the associated endophytes may play a role in enhancing the survival of the host trees by producing effective metabolites with interesting bioactivities. Moreover, we decided to use UPLC–Orbitrap–MS as UPLC enables fast and sensitive analyses with ultra-high performance for complex samples, and Orbitrap has a high resolving power and is thereby suitable for the accurate mass measurements and characterization of compounds.

## 2. Results and Discussion

### 2.1. Identification of the Endophytic Fungal Isolates

**Figure 1:**
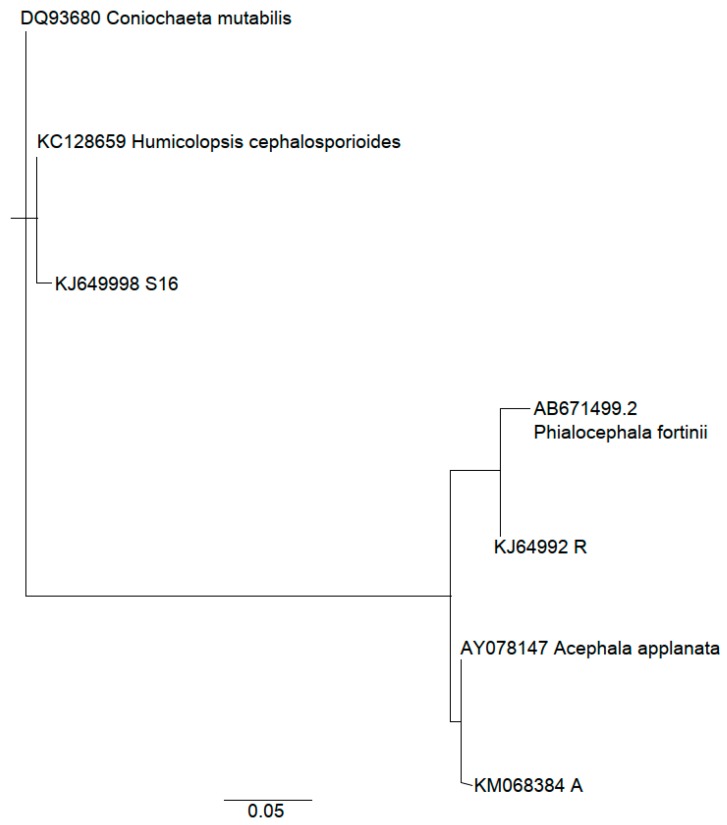
The Neighbor-Joining topology of ITS1, 5.8S and ITS2 rDNA sequences of root endophyte strains A, R and S16 from Scots pine and those obtained from GenBank. The phylogenetic tree was rooted with the *Coniochaeta mutabilis*.

The taxonomic identification of the isolated fungal strains (A, R, and S16) was made by comparing the ITS (Internal Transcribed Spacer) region with the best GenBank Blast matches (Table 1). According to the DNA sequencing of ITS1, 5.8S and ITS2 rDNA regions, A and R strains belong to PAC. ITS region of the S16 strain matched with the two species *Humicolopsis cephalosporioides* and *Coniochaeta mutabilis*. The alignments of the S16 strain together with its best GenBank matches are presented in Supplementary Figure S1. PAC species cannot be reliably separated using only the ITS regions which emphasizes the need for complementing sequencing methods in order to validate the results [11]. However, the strain A had 100% identity together with *Acephala applanata* strain (AY078147.1), whereas strain R was more similar with *Phialocephala fortinii* strains (see alignments in Supplementary Figure S2). *Humicolopsis cephalosporioides* and *Coniochaeta mutabilis* can be considered as DSE-like fungi [19]. Previously, *Coniochaeta* were considered as *Lecytophora* sp. [20]. The phylogenetic tree (Figure 1) is rooted with *Coniochaeta mutabilis*. Fungal species used in this study are to be joined into the microbe and yeast library collection of Natural Resources Institute Finland.

### 2.2. Identification of the Metabolites

We were able to identify 220 metabolites from three fungal extracts (Table 2) and 98 compounds were left unidentified (Supplementary Table S1). Identification was mainly based on the exact masses and the molecular formula observed. It was performed with Thermo Compound Discoverer software and SciFinder Scholar database with the substance role Occurrence and the highest number of references to scale down possible compound hits. Compound Discoverer hits were also run through SciFinder database to verify that the identified products have been found in natural sources. The references shown in Table 2 were used to complement the identification and additionally 39 authentic standards were examined. Metabolites, whose presence and identity were verified with authentic standards, are marked with the reliability of three stars. The majority of the identified metabolites belonged to the group of amino acids, dipeptides and peptides. In addition to the main metabolites, minor ones were also present but the intensity limit of the peaks in the ion chromatogram was set at 1 × 10^7^, and the peaks with intensities lower than that were not included.

#### 2.2.1. Amino Acids, Dipeptides and Peptides

**Figure 2:**
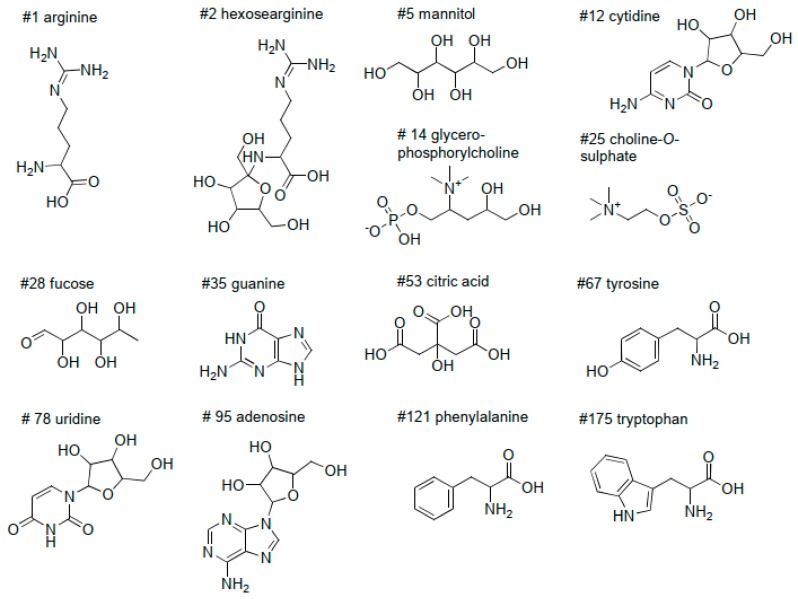
Structures of some of the identified compounds with #ID from Table 2.

Amino acids, dipeptides and tripeptides are the most abundant group of identified metabolites in Table 2, as can be expected with water extraction. With the used *m/z* range 150–2000, we were able to detect five amino acids: arginine, valine, tyrosine, phenylalanine and tryptophan (Figure 2). All except valine were verified with authentic standards and their retention time order is similar to that found in the literature [23,24,55]. Valine was detected by the [2M + H]^+^ ion as its molecular weight is too low for the scan range used. Arginine was found to be the most dominant compound and it was detected with multiple retention times and the intensities up to 1 × 10^9^ in the mass spectrum. It was also detected as a degradation product of many peptides or other structures with arginine backbone, which explains the multiple retention times. Amino acids formed potassium adducts and [2M + H]^+^ and [2M − H]^−^ ions were also observed for them. In addition, arginine was detected by [3M + H]^+^ and [3M − H]^⁻^ ions and tryptophan with ammonium fragment ions. 

**Figure 3:**
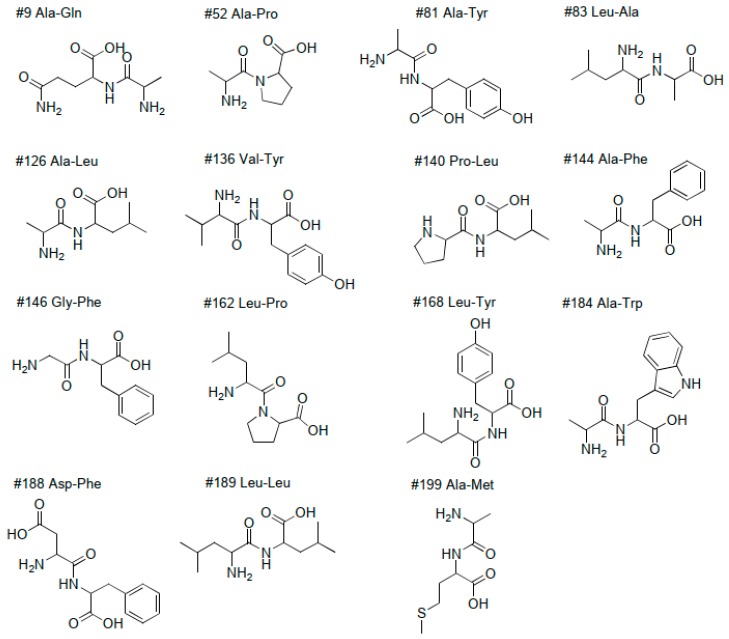
Dipeptides, whose presence was verified with authentic standards and #ID from Table 2.

Dipeptides were the most abundant class of compounds in our samples (Table 2) with 122 possible identifications, which represents 55% of the identified metabolites. Out of the dipeptides, 16 were verified with autentic standards (Figure 3). In addition, three tentative identifications of cyclic dipeptides were made using databases and listed references in Table 2: Cyclo(Glu-Tyr); Cyclo(3-OH-Pro-Tyr) and Cyclo(Glu-Leu) or Cyclo(Glu-Ile). Plant associated microorganisms are known to produce a variety of N-containing compounds, such as cyclic peptides and peptides [6,83,84,101].

Furthermore, 29 tripeptides and other oligopeptides were tentatively identified in our samples (Table 2) together with 14 amino acid derivatives. Small peptides and amino acids have also been identified from the tubers of *Pinellia ternate* roots [26] and some of the findings confirm the retention order of the compounds identified by databases and literature in our study. For some compounds, additional ions were detected. Ammonium adducts were typical for aceglutamide and acetylcitrulline type compounds and the sodium adduct was detected for acetylleucine and acetylisoleucine. Glutathione yielded an additional doubly charged ion. It is a peptide produced in response to several stress situations in endophytic microbes [17,65]. Its retention order in comparison to acetylcarnitine, tyrosine, adenosine, phenylalanine and tryptophan is the same as reported by Ibanez et al. [55]. Additional ions of tryptophan and its ammonium fragment were detected for acetyltryptophan and for Trp-Ala dipeptide, which strengthens the tentative identifications. The retention order of arginine, dimethylarginine, tyrosine and tryptophan was similar to that found in Liang et al. [25]. Dimethylarginine was also detected with ESI–MS by Gamal–Eldin et al. [33]. In addition, methionine and its derivatives, such as acetylmethionine have an important role in the biochemistry of plan tissues [86]. 

The proteins and enzymes produced by the endophytes have been reported to increase thermostability, pH-stability, UV tolerance and products with activity against pathogenic microorganisms [6]. Peptides produced by the endophytes have also been searched for new antibiotic compounds and other bioactive properties [102,103]. Antimicrobial peptides and proteins have been found to be biosynthesized immediately in response to pathogenic microorganism assault [104,105,106]. In this study, we detected large amounts of arginine, which is commonly used as nitrogen storage because it has the highest nitrogen to carbon ratio out of all 21 proteinogenic amino acids [107]. Nitrogen is often a limiting resource for the plant growth since it is needed for nucleic acid and protein synthesis. Arginine is used in the production of nitric oxide and polyamines in plants as well and both play a crucial role in the responses to abiotic and biotic stress [107]. One of the cyclic dipeptides found, Cyclo(3-OH-Pro-Tyr) was reported with acaricidal activity against *Tetranychus urticae* and cyclic dipeptides have been described being active towards plant pathogens [84,108].

#### 2.2.2. Opine Amino Acids

Four possible identifications in Table 2 were opines or N^2^-(1-carboxyethyl)-amino acids. Heliopine is a conjugate of glutamine and pyruvate, whereas rideopine is a product of the reductive amination of polyamine putrescine with α-ketoglutaric acid [34]. Lysopine is a condensation product of lysine and pyruvate [43]. Heliopine, rideopine and lysopine have all been detected from crown gall tumours produced by rhizosphere bacteria *Agrobacterium tumefaciens* [34,43]. Valinopine has been detected from a poisonous mushroom *Clitocybe acromelalga* and is suggested being a fungal toxin [92]. In addition, we were able to observe a compound tentatively identified as saccharopine, which is a precursor of lysine in the fungal α-aminoadipate pathway [109]. However, the intensities were under 1 × 10^7^ and, thus, it was not included in Table 2.

#### 2.2.3. Amino Acid Quinones and Amadori Compounds

Abenquine C or its enantiomer and Abenquine B1 and B2 are tentatively identified amino acid quinone derivatives in Table 2. Abenquine C or *N*-[4-(acetylamino)-3,6-dioxo-1,4-cyclo-hexa- dien-1-yl]-l-valine and *N*-[4-(acetylamino)-3,6-dioxo-1,4-cyclohexadien-1-yl]-leucine (Abenquine B1) and -isoleucine (Abenquine B2) have been isolated from the rhizosphere bacteria *Streptomyces* sp. strain DB634 [69,70]. Because more than one possible amino acid quinone masses were detected and they have been isolated from the rhizosphere, there is a possibility that root-colonizing fungi of the rhizosphere could produce these metabolites. However, the confidence level is putative identification.

Furthermore, Amadori compounds were detected. They are Maillard reaction products where amino acid is attached to a pentose or hexose sugar. Namely, hexosearginine, hexosevaline, pentoseproline, hexoseaminobutyric acid and deoxyhexosethreonine were among the tentatively identified compounds in Table 2. Out of these, the presence of hexosearginine (Figure 2) was verified with a synthetic reference compound (purity 99%) [110]. Additionally, hexosearginine’s fragmentation into arginine was detected in UPLC-MS. Amadori compounds have previously been identified from fungal cultures [28] and characteristic ions similar to our findings have also been detected by Davidek et al. [93] and Wang et al. [29]. Hexoseaminobutyric acid was detected with a shorter retention time than the hexose sugar structure as also shown by Lamberts et al. [42]. Additionally, deoxyhexose amino acids have been previously detected from eukaryotic cells [79].

#### 2.2.4. Cholines

Discovered cholines presented in Table 2 are choline-*O*-sulphate and glycerophosphorylcholine (Figure 2). Choline-*O*-sulphate has been found in relatively large amounts in fungal mycelia and has been suggested to act as a storage of sulphur, which is an essential metabolite for growth in filamentous fungi [41,44]. Glycerophosphorylcholine is a part of phosphatidylcholine, which is a type of phospholipid in lecithin. Lecithin is a major component of the phospholipid membrane also found in plant tissues [41]. The occurrence of both of these compounds was confirmed with authentic standards and they were observed as potassium adducts in the positive ionization. Glycerophosphorylcholine retention time with respect to arginine, tyrosine, adenosine and tryptophan was same as found by Liang et al. [25].

#### 2.2.5. Nucleobases, Nucleosides, Nucleotides, and their Derivatives 

Out of nucleobases, we were able to detect guanine and isoguanine or oxyadenine, which are the only ones with molecular mass over 150 Da (Table 2). The identification of guanine was confirmed by an authentic standard (Figure 2). Isoguanine is a purine analog, which is formed as a result of direct oxidation of adenine [71,72].

Nucleosides contain a nucleobase with a pentose sugar unit: ribose or deoxyribose. Cytidine, pseudouridine, uridine, adenosine, guanosine isomer, deoxyguanosine, methylthymidine, and deoxythymidine (Table 2) were tentatively identified, and the presence of cytidine, uridine and adenosine was verified with authentic standards (Figure 2). Nucleosides were discovered to form formate adducts and [2M − H]^−^ cluster ions in negative ESI, and guanosine isomer also yielded a fragment ion responding to the detachment of pentose sugar unit. Methylthymidine has been used as an indicator of microbial presence in wastewaters [78].

Nucleotides are nucleosides joined with at least one phosphate group. We were able to tentatively identify adenosine monophosphate (AMP) or deoxyguanosine monophosphate (dGMP), cyclic uridine monophosphate (cUMP), deoxyribose adenosine monophosphate (dAMP), cyclic adenosine diphosphate ribose (cyclic ADP-ribose), cyclic guanosine monophosphate (cGMP) and two exact masses and molecular formulae corresponding to dinucleotides (Table 2). The dinucleotides exhibited a UV maximum at 258–261 nm, which in addition to the shape of the UV spectrum correlates with the literature [111]. The absorption maximum in our study was, however, broader and continued until 300 nm, which is likely caused by other compounds eluting simultaneously. Cyclic nucleotides are used as signaling metabolites in almost all organisms and they regulate a vast number of cellular processes [59,60,61,62]. The presence of the main fragment ion at *m/z* 152.1 was also detected with cGMP as reported in the literature [59]. ADP-ribosyl groups are formed on target proteins as a response to DNA damage and poly(ADP-ribose) polymerase enzyme homologs, which catalyze the reaction, have also been found in fungi [66]. In addition, nicotinamide riboside and nicotinamide adenine dinucleotide (NAD) were tentatively identified. NAD produced a fragment ion at *m/z* 540.1 in the negative ESI mode corresponding to the cleavage of nicotinamide. The retention order of the above mentioned metabolites was similar to that found in the literature [25,26,36,37,38,39,40,48,51,52,53,55,63,64,85]. Shiao et al. [35] detected nucleosides and nucleobases from the pathogenic fungus *Cordyceps sinensis* and their retention order is same as ours.

Additionally, sugar-nucleotides, such as uridine diphosphate (UDP)-glucose and UDP-galactose as well as UDP-galactosamine and UDP-glucosamine, were discovered (Table 2). UDP-glucosamines and UDP-galactosamines are important precursors of the bacterial and fungal cell wall [49]. Sugar nucleotides are donors of sugar groups in the biosynthesis of glycosides, polysaccharides and glycoconjugates, and they are abundant in microorganisms and plants [46]. They also possess many important roles in fungi [47,54].

#### 2.2.6. Siderophores

One exact mass corresponding to *cis*- and/or *trans*-fusarinine siderophore was found with two retention times (Table 2). The *cis-* and *trans*-fusarinine backbones are very common in many fungal siderophores [75,76]. Siderophores are low molecular weight compounds that are used for iron uptake and storage and they have, for example, been found to have importance in the maintenance of plant–fungi symbioses [74,77]. Fungi and other microorganisms have been found to produce siderophores under aerobic growth conditions, where low iron availability is detected [75]. Iron is essentially required for the growth and proliferation in both bacteria and fungi and siderophores provide cells with nutritional iron [102]. In DSE fungi, it was found that these species have the ability to acidify the environment and produce siderophores to increase the micronutrient uptake to both members of the symbiont, indicating the association to be mutualistic rather than pathogenic [73]. Fusarinine monomers where also discovered with characteristic formic acid and water fragment ions.

#### 2.2.7. Other Common Metabolites

Pentonic and hexonic acids in Table 2 were identified by their exact masses and molecular formulae according to Sun et al. [26], where they had further identified the species being ribonic and gulonic acids using MS/MS data. Glycerophosphoinositol is closely related to glycerophosphorylcholine. It is found in both plants and fungi and is a major deacylation product of lipid metabolism [30,31,55]. In addition, as with cholines, the potassium adduct was observed for it.

Acetyl coenzyme A is a central carbon and energy cycle metabolite, which is bulky and amphibilic and, thus, cannot readily transverse biological membranes. Acetylcarnitine is used in fungi to transport the acetyl unit [50]. The retention order of acetylcarnitine in relation to tyrosine, glutathione dimer, adenosine, phenylalanine and tryptophan is also similar to that found by Ibanez et al. [55].

Isocitrate and citric acid are isomers with the same molecular formula as well as methylisocitric acid and methylcitric acid. Citric acid and isocitrate are both important intermediates in the Krebs cycle, which is the metabolic route to produce energy to the eukaryotic cells, such as in plants and fungi. The presence of citric acid was confirmed with an authentic standard (Figure 2). In addition, we observed potassium adducts with both citric acid and isocitrate, which strengthened the tentative identification of isocitrate. Methylcitrate cycle catabolizes propionate in yeast and filamentous fungi [57]. Propionate is produced during the catabolism of amino acids and fatty acid oxidation in higher eukaryotes and is toxic, thus, inhibiting the cell growth [112]. Methylcitrate cycle metabolizes it into pyruvate, which can be used as a source of carbon [58]. Methylcitric acid and methylisocitric acid are important intermediates in this cycle.

#### 2.2.8. Sugars, Sugar Alcohols, Disaccharides

The presence of mannitol and fucose (Table 2) was confirmed using authentic standards (Figure 2). Mannitol is widely distributed in filamentous fungi and stored in the fungal hyphae as a carbon source [32]. Fucose appears to represent a prominent feature in protein-linked glycans in the fungal kingdom [45]. Additionally, disaccharides, such as the one isolated from pathogenic fungal species *Claviceps africans*, with fructofuranose and arabinose backbone [56] were found. Deoxyhexoses, then again, are produced in fungi by pyranose oxidases, which have been reported among lignin-degrading fungi [68] for example. Deoxyhexose yielded fragment ions corresponding to the cleavage of water, whereas dehydrohexose structure was detected by its sodium and ammonium adducts and by [2M + H]^+^ and [2M − H]^−^ ions. Dehydrohexose has also been previously reported from evergreen trees [67]. 

#### 2.2.9. Endophyte or Plant Metabolites

Phomone A and B are enantiometric α-pyrone dimers isolated from the endophytic fungus *Phoma* sp. YN02-p-3 [81,82]. Blumeoside C, which is an iridoid glucoside isolated from *Fagraea blumei* [80] has the same molecular formula. Cuendet et al. [80] discovered that Blumeoside A elutes later than Blumeoside C, which is in accordance with our findings (Table 2).

Asperulosidic acid and its stereoisomer were isolated from the plant *Hedyotis diffusa* using water extraction [89]. According to Friscic et al. [90], asperulosidic acid elutes later than mannitol using reversed-phase liquid chromatography as in our study (Table 2). Asperulosidic acid has also been isolated from *Vernonia cinerea* with ethanol [91] and its structural isomers from *Morinda coreia* and *Saprosma scortechinii* with methanol [87,88].

Furthermore, we were able to find exact masses corresponding to orsellinic acid esters, which have been isolated from the endophytic *Chaetomium* sp. fungus [16,95,96,97]. However, orsellinic acid ester Globosumone B was not included in the Table 2 because of the chosen intensity limit 1 × 10^7^.

Two possible triterpene saponin structures were obtained with the molecular formula C_35_H_50_O_12_, one could be Dianthosaponin F, which has been isolated from *Dianthus japonicus* with methanol [98], and the other Celosin F, which has been isolated from *Celosia argentea* with 50% ethanol [99].

Linamarin is a cyanogenic glucoside isolated from the cassava (*Manihot esculenta*) roots [94]. Ramulosin derivatives have been previously isolated from endophytic fungi *Nigrospora* sp. Present in the branches of *Garcinia nigrolineata* tree [16,100].

### 2.3. Metabolites in Fungal Extracts

**Figure 4:**
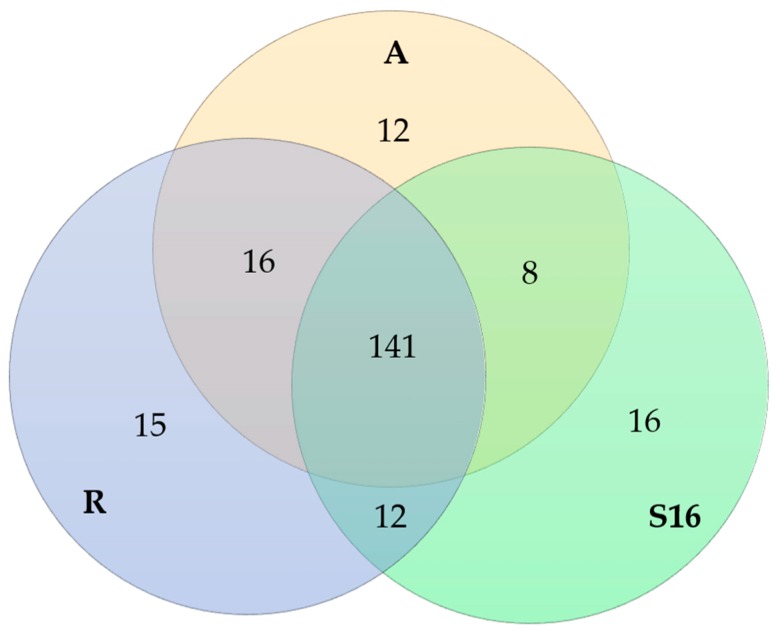
A Venn diagram of the 220 identified metabolites and how they are distributed among the fungal species A (*A. applanata*), R (*P. fortinii*) and S16 (*H. cephalosporioides* or *C. mutabilis*).

We conducted a qualitative study on the screening and identification of the water-soluble metabolites from the endophytic fungi extracts. There was a high number of primary metabolites in the aqueous extracts as expected. The number of identified metabolites was almost the same with all of the fungal species, and a majority of the metabolites, 141 compounds, were detected from all of the fungal extracts (Figure 4). From the extract A (*A. applanata*), we identified 177 metabolites and out of these 12 metabolites were exclusively found in fungus A (Figure 4). These twelve were all dipeptides or peptides except the nucleoside derivative 5-methoxycarbonylmethyluridine (Table 2). From the extract of fungus R (*P. fortinii*) 184 metabolites were identified and 15 of the metabolites were found only in this extract. These included fucose, guanine, acetylcitrulline, disaccharides, dinucleotides, acetylleucine or acetylisoleucine, the endophytic fungi metabolite orsellinic acid ester and the plant metabolites blumeoside A and asperulosidic acid as well as dipeptides and peptides. From the extract of fungus S16 (*H. cephalosporioides* or *C. mutabilis*) we identified 177 metabolites and 16 of these were found in the S16 extract only. These included hexosevaline, pseudouridine, acetylglutamic acid, cUMP, NAD and saponin as well as Ala-Glu or Glu-Ala or heliopine and other dipeptides and peptides.

In this study, we identified a large number of water-soluble metabolites that may make a contribution to nutrient intake or stress-resistance of the host plant. Many of the identified compounds have been previously reported possessing interesting bioactivities. Thus, the bioactive properties of the fungal isolates and their sub-fractions are to be investigated in the future for their antimicrobial and antioxidant properties to evaluate their potential for the host plant vitality and other applications. To our knowledge, this is the first time that metabolic profiling is conducted on these Scots pine associated endophytic fungi species using water extracts. Thus, this work offers valuable reference about the metabolites of similar endophytes, which are to be discovered in the future.

## 3. Materials and Methods 

### 3.1. Reagents

Ala-Phe, Ala-Tyr, Asp-Phe (methyl ester), Leu-Leu (acetate), Leu-Pro (hydrochloride), Phe-Ala, Pro-Gly, Pro-Leu, Tyr-Ala, Val-Tyr, guanine, cytidine, uridine, guanosine (hydrate), β-(–)-adenosine, d-mannitol, l-tyrosine, l-arginine (hydrochloride), l-phenylalanine, l-(–)-fucose and *trans*-3-indoleacrylic acid were obtained from Sigma-Aldrich (Saint Louis, MO, USA) each with purity ≥98%. l-α-Glycerophosphorylcholine (purity 99%) and Choline-*O*-sulphate (D13, purity 98%) were purchased from Carbosynth Limited, Compton, UK. DL-Ala-DL-Leu (purity ≥95%), DL-Ala-DL-Met, DL-Ala-DL-Val, l-Ala-l-Gln (HPLC grade), l-Ala-l-Trp, l-Ala-l-Pro (purity >96%), Gly-l-Ile (purity >99%), Gly-DL-Leu, Gly-l-Phe (HPLC grade), Gly-l-Pro (HPLC grade), DL-Leu-Gly, l-Leu-l-Tyr, DL-Leu-DL-Val, l-Leu-l-Ala (hydrate), and Salicin (HPLC grade) were obtained from TCI Europe, Zwijndrecht, Belgium, with purity >98% unless specified. l-(–)-tryptophan (purity >99%) was purchased from Acros Organics/Thermo Fisher Scientific, Waltham, MA, USA. Hydrogen peroxide and citric acid (monohydrate, purity >99%) were obtained from Merck KGaA, Darmstadt, Germany, and ethanol from Altia, Helsinki, Finland.

### 3.2. Endophytic Fungi Isolation and Identification

The fungal endophytes were originally isolated from the roots of eight-year-old Scots pine (*Pinus sylvestris* L.) trees grown on a drained peatland forest site in western Finland. Scots pine roots were washed and the root tips were examined under a dissecting microscope. The root tips showing signs of potential fungal association or mycorrhizal features were selected for surface sterilization with a short bath in 70% ethanol and 30% H_2_O_2_ and followed by laying on sterile Petri dishes on agar. The pure cultures of the fungus mycelium were cultivated on a solid Hagem agar [113] on Petri dishes.

The species of the fungus isolates were identified with molecular methods. DNA from the fungus mycelium was extracted using E.Z.N.A. Fungal DNA Mini Kit (Omega bio-tek, Norcross, GA, USA) according to the manufacturer’s instructions. The nucleotide sequence of the Internal Transcribed Spacer (ITS) region of fungal ribosomal DNA (rDNA) was analysed in Macrogen Inc. (Amsterdam) from polymerase chain reaction (PCR) product amplified with ITS1 and ITS4 primer pair [114]. The reaction mixture of 50 µL included, 10x enzyme buffer (Biotools B&M Labs, S.A. Madrid, Spain), 0.5 µM each primers, 0.2 µM dNTP mix, DNA Polymerase (5 U/µL) (Biotools B&M Labs, S.A. Madrid, Spain) and 1 µL DNA template. The PCR were performed with the following conditions: initial incubation at 94 °C for 5 min followed by 25 cycles of 1 min at 94 °C, 1 min at 58 °C and 1.5 min at 72 °C. Sequences with a similarity of >99% to ITS1, 5.8S and ITS2 rDNA regions were considered as identical species [115]. ITS1, 5.8S and ITS2 regions were extracted from the fungal ITS sequences and the cleaned sequences were used for BLAST searches against GenBank/NCBI to provide taxonomic identification. The best matches from GenBank were aligned and a phylogenetic tree was generated in Geneious 6.0.6 using the Neighbor-Joining analysis (Figure 1). The sequences were deposited in GenBank with accession numbers KM068384, KJ649992 and KJ649998.

### 3.3. Fungal Extract Preparation

The fungal mass was collected from the surface of a cellophane membrane on agar [113] with a scalpel, stored at −80 °C, and ground in a mortar before adding to sealed, sterile and previously weighed polypropylene test tubes (BD Falcon™, VWR International Oy, Helsinki, Finland). The extraction was executed with boiling deionized and filtered (0.2 µm, Nylon 66 Filter Membrane from Supelco by Sigma–Aldrich Co, Saint Louis, MO, USA) water. The fungal mass was mixed with equal amount of boiling deionized and filtered (0.45 µm Nylon membrane, Supelco Analytical/Sigma Aldrich, Saint Louis, MO, USA) water (1 mL = 1 g) by vortexing. Extraction tubes were then shaken in +95–100 °C water bath (SW22, JULABO Labortechnik GmbH, Seelbach, Germany) for 15 minutes after which the tubes were cooled in an ice bath before vortexing again. Extraction tubes were then centrifuged at +4 °C and 8200 g for 10 minutes (Eppendorf Centrifuge 5804R, Hamburg, Germany). The supernatants were collected into new polypropylene tubes and centrifuged again with the same settings. Finally, the supernatants were filtered through nylon syringe filters (0.2 µm, Cronus Filter from SMI–LabHut Ltd., Gloucester, UK) to new polypropylene tubes. Aliquots of water without fungal material were extracted simultaneously as control samples. The pH of the extracts was measured with indicator paper (scale 1–14) and it ranged from 5.5 to 7. Extracts were dried with freeze-drying equipment (VirTis BenchTop 6K with Trivac E2, D 2,5E Vacuum pump, SP Industries, Warminster, PA, USA) before storing at −80 °C. The dried extracts were dissolved in sterile purified water before analysis.

### 3.4. UHPLC-DAD-ESI-Orbitrap-MS

After vacuum drying, 20 µL of ethanol and 980 µL of water were added to the samples. The samples were then mixed with a vortex and filtered using 0.2 µm PTFE syringe filter prior to analyses. The samples were analyzed using an ultra-high performance liquid chromatograph coupled to a photodiode array detector (UHPLC–DAD, Acquity UPLC, Waters Corporation, Milford, MA, USA) and a hybrid quadrupole-Orbitrap mass spectrometer (Q Exactive™, Thermo Fisher Scientific GmbH, Bremen, Germany). The column was Acquity UPLC^®^ BEH Phenyl (100 × 2.1 mm i.d.; 1.7 µm; Waters Corporation, Wexford, Ireland). The mobile phase consisted of (A) acetonitrile and (B) water and formic acid (99.9:0.1, The elution profile was as follows: 0–0.5 min, 0.1% A; 0.5–5.0 min, 0.1–30% A (linear gradient); 5.0–5.1 min, 30–90% A (linear gradient); 5.1–7.1 min, 90% A; 7.1–7.2 min, 90–0.1% A (linear gradient); 7.2–8.5 min, 0.1% A. The injection volume was 5 µL and flow rate 0.5 mL/min. The UV data was collected at 190–500 nm. The heated ESI source (H–ESI II, Thermo Fisher Scientific GmbH, Bremen, Germany) was operated both in negative and positive ion modes. The parameters for negative ionization were as follows: spray voltage was set at –3.0 kV, sheath gas (N_2_) flow rate at 60 (arbitrary units), aux gas (N_2_) flow rate at 20 (arbitrary units), sweep gas flow rate at 0 (arbitrary units), capillary temperature at +380 °C and S-lens RF level at 60. The parameters for positive ionization were similar, except that spray voltage was set at 3.8 kV. Orbitrap was set at a resolution of 70,000 and an automatic gain of 3 × 10^6^ was used. Masses were scanned at *m/z* 150–2000. Pierce ESI Negative Ion Calibration Solution (Thermo Fischer Scientific Inc., Waltham, MA, USA) was used to for the calibration. The data was processed with Thermo Xcalibur Qual Browser software (Version 3.0.63, Thermo Fisher Scientific Inc., Waltham, MA, USA).

### 3.5. Identification

Orbitrap data was processed using Compound Discoverer 2.1 SP1 (Thermo-Fisher Scientific, Waltham, MA, USA). The processing flow ‘Untargeted Metabolomics Workflow’ was utilized. The following general settings were used for the workflow: mass tolerance = 5 ppm, intensity threshold = 30%, S/N threshold = 3, minimum peak intensity = 1 × 10^6^, maximum element counts = 100 × C, 200 × H, 10 × N, 100 × O, 10 × S and 10 × P. The following settings were used for the peak detection: filter peaks = true, maximum peak width = 0.5 min, remove singlets = true, minimum # scans per peak = 5 and minimum # isotopes = 1. ChemSpider and KEGG databases were used for the identification. In addition, we used SciFinder Scholar database (American Chemical Society, CAS, Columbus, OH, USA) with substance role Occurrence and highest number of references to scale down possible compound hits.

## Figures and Tables

**Table 1:** The endophytic fungus isolates and NCBI information about the best match and our identification.

Strain (GenBank Accession NO.)	GenBank Accession NO. for the Best Match	Max Identity (%)/Query Coverage (%)	Our Description for the Strain	Order	Class	Phylum
A (KM068384)	AY078147.1	100/98	*Acephala applanata*	Helotiales	Leotiomycetes	Ascomycota
R (KJ649992)	AB671499.2	100/100	*Phialocephala fortinii*	Helotiales	Leotiomycetes	Ascomycota
S16 (KJ649998)	KC128659	99/98	*Humicolopsis cephalosporioides*			Ascomycota
	DQ93680	99/98	*Coniochaeta mutabilis*	Coniochaetales	Sordariomycetes	Ascomycota

**Table 2:** The identified metabolites from the aqueous endophytic fungi extracts. Metabolomics Standards Initiative (MSI) was used to define the metabolite identification confidence [21,22]. Level 1 is for confidently identified compounds, where an authentic chemical standard was analyzed under same analysis conditions. Level 2 is for putatively annotated compounds, where physicochemical properties and spectral similarities with public spectral libraries as well as listed references were used. Level 3 is for putatively annotated compound classes, where characteristic physicochemical properties or spectral similarities of compound classes are used to confirm identity. Unidentified compounds are shown in the Supplementary Table S1 and their identification is level 4: unidentified and unclassified but can be differentiated based upon spectral data. Listed references were used to confirm the identification for example by similar findings from natural sources and further justifications are described after the table. If the observed intensity of the metabolite in the total ion chromatogram is over 1 × 10^7^, metabolite is marked with x under the corresponding fungal extract. nd = not detected. RDB = ring-double bond equivalent.

				Exact Mass		Error							Fungal Extract		
#ID	RT	[M + H]^+^	[M − H]^−^	Measured	Calculated	Δm (ppm)	Molecular Formula	RDB	Compound	Class	MSI	References	A	R	S16
1	0.46	175	173	174.11199	174.11168	1.8	C_6_H_14_O_2_N_4_	2	Arginine	Amino acid	1	[23,24,25,26]	x	x	x
2	0.49	337	335	336.16316	336.16450	−4.0	C_12_H_24_O_7_N_4_	3	Hexosearginine	Amadori	1	[27,28,29]	x	x	x
3	0.51	335	333	334.06709	334.06650	1.8	C_9_H_19_O_11_P	2	Glycerophospho-inositol	Common metabolite	2	[30,31]	x	-	x
4	0.52	372	370	371.22764	371.22810	−1.2	C_15_H_29_O_4_N_7_	5	Ace-Ala-Arg-Ala-NMe	Peptide	2		x	x	x
5	0.53	183	181	182.07875	182.07904	−1.6	C_6_H_14_O_6_	1	Mannitol	Hexitol	1	[24,26,32]	x	x	x
6	0.53	246	244	245.14851	245.14879	−1.1	C_9_H_19_O_3_N_5_	3	Ala-Arg or Arg-Ala	Dipeptide	2		-	x	x
7	0.54	nd	165	166.04668	166.04774	−6.4	C_5_H_10_O_6_	1	Pentonic acid	Pentonic acid	3	[26]	x	x	x
8	0.54	nd	195	196.05748	196.05831	−4.2	C_6_H_12_O_7_	1	Hexonic acid	Hexonic acid	3	[26]	x	x	x
9	0.54	218	216	217.10586	217.10626	−1.8	C_8_H_15_O_4_N_3_	3	Ala-Gln	Dipeptide	1		-	-	x
10	0.55	203	201	202.14253	202.14298	−2.2	C_8_H_18_O_2_N_4_	2	Dimethylarginine	Amino acid derivative	2	[25,33]	x	x	x
11	0.55	219	217	218.08984	218.09027	−2.0	C_8_H_14_O_5_N_2_	3	Ala-Glu or Glu-Ala or Heliopine	Dipeptide or Opine amino acid	2	[34]	-	-	x
12	0.55	244	242	243.08558	243.08552	0.2	C_9_H_13_O_5_N_3_	5	Cytidine	Nucleoside	1	[25,26,35,36,37,38,39,40]	x	x	x
13	0.55	248	246	247.11641	247.11682	−1.7	C_9_H_17_O_5_N_3_	3	Gln-Thr or Thr-Gln	Dipeptide	2		-	-	x
14	0.55	258	nd	257.10240	257.10282	−1.6	C_8_H_20_O_6_NP	1	Glycerophosphoryl-choline	Choline	1	[25,41]	x	x	x
15	0.56	191	189	190.09479	190.09536	−3.0	C_7_H_14_O_4_N_2_	2	Ala-Thr or Thr-Ala	Dipeptide	2		x	x	x
16	0.56	235°	nd	234.15747	234.15796	−2.1	C_10_H_22_O_4_N_2_	1	Valine	Amino acid	2	[23,24,26]	-	x	x
17	0.56	253	251	252.12182	252.12224	−1.7	C_11_H_16_O_3_N_4_	6	His-Pro or Pro-His	Dipeptide	2		x	x	x
18	0.56	266	264	265.11662	265.11615	1.8	C_10_H_19_O_7_N	2	Hexoseaminobutyric acid	Amadori	3	[42]	x	x	-
19	0.57	205	203	204.11052	204.11101	−2.4	C_8_H_16_O_4_N_2_	2	Ser-Val or Val-Ser	Dipeptide	2		-	x	x
20	0.58	187	185	186.10007	186.10044	−2.0	C_8_H_14_O_3_N_2_	3	Pro-Ala	Dipeptide	2		-	-	x
21	0.58	198	196	197.07971	197.08004	−1.7	C_8_H_11_O_3_N_3_	5	Acetylhistidine	Amino acid derivative	2		-	x	x
22	0.58	219	217	218.12640	218.12666	−1.2	C_9_H_18_O_4_N_2_	2	Dipeptide^a^ or Lysopine or Rideopine	Dipeptide or Opine amino acid	3/2	[34,43]	x	x	x
23	0.58	246	244	245.13727	245.13756	−1.2	C_10_H_19_O_4_N_3_	3	Dipeptide^b^	Dipeptide	3		x	x	x
24	0.58	260	258	259.18913	259.18959	−1.8	C_12_H_25_O_3_N_3_	2	Dipeptide^c^	Dipeptide	3		x	x	x
25	0.59	184	nd	183.05615	183.05653	−2.1	C_5_H_13_O_4_NS	2	Choline-*O*-sulphate	Choline	1	[41,44]	x	x	x
26	0.60	233	231	232.10553	232.10592	−1.7	C_9_H_16_O_5_N_2_	3	Asp-Val or Val-Asp	Dipeptide	2		x	x	x
27	0.60	335	333	334.06584	334.06650	−2.0	C_9_H_19_O_11_P	2	Glycerophospho-inositol	Common metabolite	2	[30,31]	x	x	x
28	0.61	165	163	164.06831	164.06848	−1.0	C_6_H_12_O_5_	1	Fucose	Hexose	1	[45]	-	x	-
29	0.64	191	189	190.09482	190.09536	−2.8	C_7_H_14_O_4_N_2_	2	Ala-Thr or Thr-Ala	Dipeptide	2		x	x	-
30	0.64	219	217	218.12619	218.12666	−2.2	C_9_H_18_O_4_N_2_	2	Dipeptide^a^ or Lysopine or Rideopine	Dipeptide or Opine amino acid	3/2	[34,43]	-	x	x
31	0.64	219	217	218.08980	218.09027	−2.2	C_8_H_14_O_5_N_2_	3	Ala-Glu or Glu-Ala or Heliopine	Dipeptide or Opine amino acid	2	[34]	-	-	x
32	0.64	nd	565	566.05592	566.05502	1.6	C_15_H_24_O_17_N_2_P_2_	8	UDP-galactose	Nucleotide sugar	2	[46,47]	x	x	x
33	0.65	217	215	216.12187	216.12224	−1.7	C_8_H_16_O_3_N_4_	3	Acetylarginine	Amino acid derivative	2	[26]	x	x	x
34	0.66	255	nd	254.08980	254.09027	−1.8	C_11_H_14_O_5_N_2_	6	Nicotinamide riboside	Pyridine nucleoside	2	[48]	x	x	-
35	0.67	152	150	151.04916	151.04941	−1.7	C_5_H_5_ON_5_	6	Guanine	Nucleobase	1	[35,38]	-	x	-
36	0.67	247	245	246.12114	246.12157	−1.7	C_10_H_18_O_5_N_2_	3	Dipeptide^d^	Dipeptide	3		x	x	x
37	0.68	219	217	218.08995	218.09027	−1.5	C_8_H_14_O_5_N_2_	3	Ala-Glu or Glu-Ala or Heliopine	Dipeptide or Opine amino acid	2	[34]	-	-	x
38	0.68	608	606	607.08235	607.08157	1.3	C_17_H_28_O_17_N_3_P_2_	8	UDP-galactosamine	Nucleotide sugar	2	[45,49]	x	x	x
39	0.69	247	245	246.12112	246.12157	−1.8	C_10_H_18_O_5_N_2_	3	Dipeptide^d^	Dipeptide	3		x	x	x
40	0.69	288	286	287.19514	287.19574	−2.1	C_12_H_25_O_3_N_5_	3	Dipeptide^e^	Dipeptide	3		x	x	x
41	0.71	189	187	188.07948	188.07971	−1.2	C_7_H_12_O_4_N_2_	3	Aceglutamide	Amino acid derivative	2		x	x	x
42	0.72	193	191	192.02628	192.02701	−3.8	C_6_H_8_O_7_	4	Isocitrate	Common metabolite	2	[50]	x	x	x
43	0.72	280	278	279.13127	279.1318	−1.9	C_11_H_21_O_7_N	2	Hexosevaline	Amadori	2	[28,29]	-	-	x
44	0.73	246	244	245.13705	245.13756	−2.1	C_10_H_19_O_4_N_3_	3	Dipeptide^b^	Dipeptide	3		x	x	x
45	0.74	348	346	347.06239	347.06309	−2.0	C_10_H_14_O_7_N_5_P	8	AMP or dGMP	Nucleotide	2	[51,52,53]	-	x	x
46	0.74	nd	565	566.05580	566.05502	1.4	C_15_H_24_O_17_N_2_P_2_	8	UDP-glucose	Nucleotide sugar	2	[46,47,53,54]	x	x	x
47	0.77	204	nd	203.11554	203.11576	−1.1	C_9_H_17_O_4_N	2	Acetylcarnitine	Common metabolite	2	[50,55]	x	x	x
48	0.77	219	217	218.12622	218.12666	−2.0	C_9_H_18_O_4_N_2_	2	Dipeptide^a^ or Lysopine or Rideopine	Dipeptide or Opine amino acid	3/2	[34,43]	x	x	x
49	0.77	333	331	332.13261	332.13320	−1.8	C_12_H_2_O_7_N_4_	5	Tripeptide/peptide^f^	Tripeptide or peptide	3		x	x	-
50	0.78	205	203	204.11084	204.11101	−0.8	C_8_H_16_O_4_N_2_	2	Ser-Val or Val-Ser	Dipeptide	2		x	x	x
51	0.78	608	606	607.08248	607.08157	1.5	C_17_H_27_O_17_N_3_P_2_	8	UDP-glucoseamine	Nucleotide sugar	2	[46,49]	x	x	x
52	0.80	187	185	186.10017	186.10044	−1.5	C_8_H_14_O_3_N_2_	3	Ala-Pro	Dipeptide	1	[26]	x	x	x
53	0.80	193	191	192.02628	192.02701	−3.8	C_6_H_8_O_7_	4	Citric acid	Common metabolite	1	[24,26]	x	x	x
54	0.80	260	258	259.18922	259.18959	−1.4	C_12_H_26_O_3_N_3_	2	Dipeptide^c^	Dipeptide	3		x	x	x
55	0.80	315	313	314.12205	314.1213	2.4	C_11_H_22_O_10_	1	Disaccharide	Disaccharide	3	[56]	-	x	-
56	0.81	189	187	188.11563	188.11609	−2.4	C_8_H_16_O_3_N_2_	2	Dipeptide^g^	Dipeptide	3		x	x	x
57	0.81	288	286	287.19523	287.19574	−1.8	C_12_H_25_O_3_N_5_	3	Dipeptide^e^	Dipeptide	3		-	x	x
58	0.81	333	331	332.13261	332.13320	−1.8	C_12_H_20_O_7_N_4_	5	Tripeptide/peptide^f^	Tripeptide or peptide	3		x	x	-
59	0.82	219	217	218.12630	218.12666	−1.7	C_9_H_18_O_4_N_2_	2	Dipeptide^a^ or Lysopine or Rideopine	Dipeptide or Opine amino acid	3/2	[34,43]	x	-	x
60	0.84	260	258	259.15272	259.15321	−1.9	C_11_H_21_O_4_N_3_	3	Di-/Tripeptide^h^	Di-/Tripeptide	3		x	x	x
61	0.85	219	217	218.12633	218.12666	−1.5	C_9_H_18_O_4_N_2_	2	Dipeptide^a^ or Lysopine or Rideopine	Dipeptide or Opine amino acid	3	[34,43]	x	x	x
62	0.85	233	231	232.14192	232.14231	−1.7	C_10_H_20_O_4_N_2_	2	Dipeptide^i^	Dipeptide	3		x	x	x
63	0.85	260	258	259.18870	259.18959	−3.4	C_12_H_26_O_3_N_3_	2	Dipeptide^c^	Dipeptide	3		x	x	x
64	0.86	221	219	220.08778	220.08817	−1.8	C_8_H_16_O_3_N_2_S	2	Cys-Val or Val-Cys or Met-Ala	Dipeptide	2		x	x	x
65	0.86	245	243	244.06948	244.06954	−0.2	C_9_H_12_O_6_N_2_	5	Pseudouridine	Nucleoside	2	[25,35]	-	-	x
66	0.86	247	245	246.12114	246.12157	−1.7	C_10_H_18_O_5_N_2_	3	Dipeptide^d^	Dipeptide	3		x	x	x
67	0.87	182	180	181.07374	181.07389	−0.8	C_9_H_11_O_3_N	5	Tyrosine	Amino acid	1	[23,24,25,26,55]	x	x	x
68	0.87	nd	205	206.04209	206.04266	−2.8	C_7_H_10_O_7_	3	Methylisocitric acid	Common metabolite	2	[57,58]	x	x	x
69	0.89	260	258	259.15288	259.15321	−1.3	C_11_H_21_O_4_N_3_	3	Di-/Tripeptide^h^	Di-/Tripeptide	3		x	x	-
70	0.91	190	188	189.06334	189.06372	−2.0	C_7_H_11_O_5_N	3	Acetylglutamic acid	Amino acid derivative	2		-	x	x
71	0.91	233	231	232.14152	232.14231	−3.4	C_10_H_20_O_4_N_2_	2	Dipeptide^i^	Dipeptide	3		x	x	x
72	0.91	260	258	259.18907	259.18959	−2.0	C_12_H_26_O_3_N_3_	2	Dipeptide^c^	Dipeptide	3		x	x	x
73	0.93	233	231	232.10574	232.10592	−0.8	C_9_H_16_O_5_N_2_	3	Asp-Val or Val-Asp	Dipeptide	2		x	x	x
74	0.95	nd	205	206.04215	206.04266	−2.5	C_7_H_10_O_7_	4	Methylcitric acid	Common metabolite	2	[57,58]	x	x	x
75	0.95	218	216	217.10599	217.10626	−1.2	C_8_H_15_O_4_N_3_	3	Acetylcitrulline	Amino acid derivative	2		-	x	-
76	0.97	190	188	189.06359	189.06372	−0.7	C_7_H_11_O_5_N	3	Acetylglutamic acid	Amino acid derivative	2		-	-	x
77	0.97	307	305	306.02467	306.02530	−2.1	C_9_H_11_O_8_N_2_P	7	2’,3’-cUMP or 3’,5’-cUMP	Nucleotide	2	[59,60,61,62]	-	-	x
78	0.99	245	243	244.06915	244.06954	−1.6	C_9_H_12_O_6_N_2_	5	Uridine	Nucleoside	1	[35,36,38,39,63]	x	x	x
79	0.99	288	286	287.19481	287.19574	−3.2	C_12_H_25_O_3_N_5_	3	Dipeptide^e^	Dipeptide	3		x	x	x
80	0.99	332	330	331.06749	331.06817	−2.0	C_10_H_14_O_6_N_5_P	8	dAMP	Nucleotide	2	[52,53,64]	-	x	x
81	1.01	253	251	252.11056	252.11101	−1.8	C_12_H_16_O_4_N_2_	6	Ala-Tyr	Dipeptide	1		x	x	x
82	1.03	261	259	260.13679	260.13722	−1.6	C_11_H_20_O_5_N_2_	3	Dipeptide^j^	Dipeptide	3		x	x	x
83	1.04	203	201	202.13162	202.13174	−0.6	C_9_H_18_O_3_N_2_	2	Leu-Ala	Dipeptide	1		x	x	x
84	1.04	215	213	214.13141	214.13174	−1.5	C_10_H_18_O_3_N_2_	3	Pro-Val or Val-Pro	Dipeptide	2		x	x	x
85	1.08	221	219	220.08783	220.08817	−1.5	C_8_H_16_O_3_N_2_S	2	Cys-Val or Val-Cys or Met-Ala	Dipeptide	2		x	x	x
86	1.10	261	259	260.13679	260.13722	−1.6	C_11_H_20_O_5_N_2_	3	Dipeptide^j^	Dipeptide	3		x	x	x
87	1.10	664	662	663.10777	663.10912	−2.0	C_21_H_27_O_14_N_7_P_2_	15	Nicotinamide adenine dinucleotide	Dinucleotide	2	[48]	-	-	x
88	1.11	260	258	259.15306	259.15321	−0.6	C_11_H_21_O_4_N_3_	3	Di-/Tripeptide^h^	Di-/Tripeptide	3		x	-	-
89	1.17	202	nd	201.11101	201.11134	−1.6	C_8_H_15_O_3_N_3_	3	Acetylmethyl-glutaminamide	Amino acid derivative	2		x	-	-
90	1.17	403	401	402.22194	402.22268	−1.8	C_16_H_30_O_6_N_6_	5	Tripeptide/peptide^k^	Tripeptide or peptide	3		x	-	-
91	1.18	613	611	612.15080	612.15197	−1.9	C_20_H_32_O_12_N_6_S_2_	8	Glutathione dimer	Tripeptide	2	[17,55,65]	x	-	x
92	1.23	315	313	314.12193	314.12130	2.0	C_11_H_22_O_10_	1	Disaccharide	Disaccharide	3	[56]	x	x	-
93	1.24	189	187	188.11571	188.11609	−2.0	C_8_H_16_O_3_N_2_	2	Dipeptide^g^	Dipeptide	3		x	x	x
94	1.24	269	267	268.10536	268.10592	−2.1	C_12_H_16_O_5_N_2_	6	Ser-Tyr or Tyr-Ser	Dipeptides	2		x	x	x
95	1.30	268	266	267.09608	267.09676	−2.5	C_10_H_13_O_4_N_5_	7	Adenosine	Nucleoside	1	[25,26,35,36,37,38,40,55,63]	x	x	x
96	1.33	315	313	314.12166	314.12130	1.2	C_11_H_22_O_10_	1	Disaccharide	Disaccharide	3	[56]	-	x	-
97	1.33	542	540	541.05998	541.06111	−2.1	C_15_H_21_O_13_N_5_P_2_	11	Cyclic ADP-ribose	Nucleotide	2	[66]	x	x	-
98	1.40	179	177	178.04742	178.04774	−1.8	C_6_H_10_O_6_	2	Dehydrohexose	Dehydro-hexose	3	[67]	x	x	x
99	1.42	221	219	220.08820	220.08817	0.1	C_8_H_16_O_3_N_2_S	2	Ala-Met	Dipeptide	1		-	-	x
100	1.47	346	344	345.04695	345.04744	−1.4	C_10_H_12_O_7_N_5_P	9	2’,3’-cGMP or 3’,5’-cGMP	Nucleotide	2	[25,38,51,59,60,61,62]	x	x	x
101	1.48	219	217	218.12633	218.12666	−1.5	C_9_H_18_O_4_N_2_	2	Dipeptide^a^ or Lysopine or Rideopine	Dipeptide or Opine amino acid	2/3	[34,43]	x	x	x
102	1.50	267	265	266.12611	266.12666	−2.1	C_13_H_18_O_4_N_2_	6	Dipeptide^l^	Dipeptide	3		x	x	x
103	1.50	284	282	283.09056	283.09167	−3.9	C_10_H_13_O_5_N_5_	7	Guanosine isomer	Nucleoside	2	[26,35,37,39]	x	x	x
104	1.54	346	344	345.04701	345.04744	−1.2	C_10_H_12_O_7_N_5_P	9	2’,3’-cGMP or 3’,5’-cGMP	Nucleotide	2	[25,38,51,59,60,61,62]	x	-	x
105	1.57	165	163	164.06821	164.06848	−1.6	C_6_H_12_O_5_	1	Deoxyhexose	Deoxyhexose	3	[68]	x	x	-
106	1.61	246	244	245.13713	245.13756	−1.7	C_10_H_19_O_4_N_3_	3	Dipeptide^b^	Dipeptide	3		x	x	x
107	1.64	219	217	218.12628	218.12666	−1.7	C_9_H_18_O_4_N_2_	2	Dipeptide^a^ or Lysopine or Rideopine	Dipeptide or Opine amino acid	3/2	[34,43]	x	x	x
108	1.64	267	265	266.12620	266.12666	−1.7	C_13_H_18_O_4_N_2_	6	Dipeptide^l^	Dipeptide	3		x	x	x
109	1.67	260	258	259.15294	259.15321	−1.0	C_11_H_21_O_4_N_3_	3	Di-/Tripeptide^h^	Di-/Tripeptide	3		x	x	x
110	1.68	294	292	293.13692	293.13756	−2.2	C_14_H_19_O_4_N_3_	7	Di/tripeptide^m^	Di-/Tripeptide	3		x	x	x
111	1.69	233	231	232.14200	232.14231	−1.3	C_10_H_20_O_4_N_2_	2	Dipeptide^i^	Dipeptide	3		x	x	x
112	1.71	281	279	280.10530	280.10592	−2.2	C_13_H_16_O_5_N_2_	7	Abenquine C or enantiomer	AA quinone	2	[69,70]	x	x	x
113	1.72	189	187	188.11572	188.11609	−2.0	C_8_H_16_O_3_N_2_	2	Dipeptide^g^	Dipeptide	3		x	x	x
114	1.74	152	150	151.04925	151.04941	−1.0	C_5_H_5_ON_5_	6	Isoguanine or Oxyadenine	Nucleobase	2	[71,72]	-	x	x
115	1.74	246	244	245.13727	245.13756	−1.2	C_10_H_19_O_4_N_3_	3	Dipeptide^b^	Dipeptide	3		x	x	x
116	1.74	279	277	278.12642	278.12666	−0.9	C_14_H_18_O_4_N_2_	7	Pro-Tyr or Tyr-Pro	Dipeptide	2		x	x	x
117	1.74	288	286	287.19527	287.19574	−1.6	C_12_H_25_O_3_N_5_	3	Dipeptide^e^	Dipeptide	3		-	-	x
118	1.75	261	259	260.13698	260.13722	−0.9	C_11_H_20_O_5_N_2_	3	Fusarinine monomer	Siderophore	2	[73,74,75,76,77]	x	x	x
119	1.75	268	266	267.09639	267.09676	−1.4	C_10_H_13_O_4_N_5_	7	Deoxyguanosine	Nucleoside	2	[35]	x	x	x
120	1.75	318	316	317.15840	317.15869	−0.9	C_13_H_23_O_6_N_3_	4	Tripeptide/peptide^n^	Tripeptide/ peptide	3		x	x	x
121	1.76	166	164	165.07893	165.07898	−0.3	C_9_H_11_O_2_N	5	Phenylalanine	Amino acid	1	[23,24,26,55]	x	x	x
122	1.76	189	187	188.11574	188.11609	−1.8	C_8_H_16_O_3_N_2_	2	Dipeptide^g^	Dipeptide	3		-	-	x
123	1.76	260	258	259.15306	259.15321	−0.6	C_11_H_21_O_4_N_3_	3	Di-/Tripeptide^h^	Di-/Tripeptide	3		x	x	x
124	1.78	257	255	256.10554	256.10592	−1.5	C_11_H_16_O_5_N_2_	5	Methylthymidine	Nucleoside derivative	2	[78]	x	x	x
125	1.78	266	264	265.11577	265.11615	−1.4	C_10_H_19_O_7_N	2	Deoxyhexose-threonine	Amadori	3	[79]	x	x	-
126	1.80	203	201	202.13164	202.13174	−0.5	C_9_H_18_O_3_N_2_	2	Ala-Leu	Dipeptide	1		x	x	x
127	1.80	260	258	259.15303	259.15321	−0.7	C_11_H_21_O_4_N_3_	3	Di-/Tripeptide^h^	Di-/Tripeptide	3		x	x	x
128	1.82	189	187	188.11586	188.11609	−1.2	C_8_H_16_O_3_N_2_	2	Dipeptide^g^	Dipeptide	3		x	x	x
129	1.82	215	213	214.13157	214.13174	−0.8	C_10_H_18_O_3_N_2_	3	Pro-Val or Val-Pro	Dipeptide	2		x	x	x
130	1.82	233	231	232.14207	232.14231	−1.0	C_10_H_20_O_4_N_2_	2	Dipeptide^i^	Dipeptide	3		x	x	x
131	1.85	307	305	306.02538	306.02530	0.3	C_9_H_11_O_8_N_2_P	7	2’,3’-cUMP or 3’,5’-cUMP	Nucleotide	2	[59,60,61,62]	x	x	x
132	1.86	260	258	259.15312	259.15321	−0.3	C_11_H_21_O_4_N_3_	3	Di-/Tripeptide^h^	Di-/Tripeptide	3		x	-	x
133	1.86	348	346	347.16926	347.16925	0.0	C_14_H_25_O_7_N_3_	4	Tripeptide^o^	Tripeptide	3		x	-	-
134	1.87	249	247	248.11934	248.11947	−0.5	C_10_H_20_O_3_N_2_S	2	Met-Val or Val-Met	Dipeptide	2		x	x	x
135	1.89	261	259	260.13682	260.13722	−1.5	C_11_H_20_O_5_N_2_	3	Fusarinine monomer	Siderophore	2	[73,74,75,76,77]	x	x	x
136	1.89	281	279	280.14177	280.14231	−1.9	C_14_H_20_O_4_N_2_	6	Val-Tyr	Dipeptide	1		x	x	x
137	1.89	295	293	294.12105	294.12157	−1.8	C_14_H_18_O_5_N_2_	7	Peptide type compound^p^	Dipeptide or peptide derivative	3	[69,70]	x	x	x
138	1.90	318	316	317.15846	317.15869	−0.7	C_13_H_23_O_6_N_3_	4	Tripeptide/peptide^n^	Tripeptide/ peptide	3		x	-	-
139	1.90	317	315	316.09129	316.09067	2.0	C_12_H_16_O_8_N_2_	7	5-methoxycarbonyl-methyluridine	Nucleoside derivative	2	[39,40]	x	-	-
140	1.91	229	227	228.14731	228.14739	−0.3	C_11_H_20_O_3_N_2_	3	Pro-Leu	Dipeptide	1		x	x	x
141	1.91	247	245	246.12143	246.12157	−0.6	C_10_H_18_O_5_N_2_	3	Dipeptide^d^	Dipeptide	3		x	x	x
142	1.95	249	247	248.11934	248.11947	−0.5	C_10_H_20_O_3_N_2_S	2	Met-Val or Val-Met	Dipeptide	2		x	x	x
143	1.96	231	229	230.16318	230.16304	0.6	C_11_H_22_O_3_N_2_	2	Dipeptide^q^	Dipeptide	3		x	x	x
144	1.96	237	235	236.11595	236.11609	−0.6	C_12_H_16_O_3_N_2_	6	Ala-Phe	Dipeptide	1		x	x	x
145	1.96	348	346	347.16933	347.16925	0.2	C_14_H_25_O_7_N_3_	4	Tripeptide^o^	Tripeptide	3		x	x	-
146	1.99	223	221	222.10043	222.10044	0.0	C_11_H_14_O_3_N_2_	6	Gly-Phe	Dipeptide	1		x	x	x
147	1.99	246	244	245.13786	245.13756	1.2	C_10_H_19_O_4_N_3_	3	Dipeptide^b^	Dipeptide	3		x	-	-
148	2.02	541	539	540.14854	540.14791	1.2	C_24_H_28_O_14_	11	Phomone A or B or Blumeoside C	Endophyte or plant metabolite	2	[80,81,82]	-	x	x
149	2.06	355	353	354.15419	354.15394	0.7	C_15_H_22_O_6_N_4_	7	Peptide^r^	Peptide	3		-	-	x
150	2.07	581	579	580.15391	580.15436	−0.8	C_20_H_25_O_9_N_10_P	15	Dinucleotide	Dinucleotide	3		-	x	-
151	2.08	229	227	228.14738	228.14739	0.0	C_11_H_20_O_3_N_2_	3	Ile-Pro or Pro-Ile	Dipeptide	2		x	x	x
152	2.09	231	229	230.16290	230.16304	−0.6	C_11_H_22_O_3_N_2_	2	Dipeptide^q^	Dipeptide	3		x	x	x
153	2.10	247	245	246.12100	246.12157	−2.3	C_10_H_18_O_5_N_2_	3	Dipeptide^d^	Dipeptide	3		x	x	x
154	2.10	306	304	305.13731	305.13756	−0.8	C_15_H_19_O_4_N_3_	8	Thr-Trp or Trp-Thr	Dipeptide	2		x	x	x
155	2.10	581	579	580.15428	580.15436	−0.1	C_20_H_25_O_9_N_10_P	15	Dinucleotide	Dinucleotide	3		-	x	-
156	2.14	318	316	317.15843	317.15869	−0.8	C_13_H_23_O_6_N_3_	4	Tripeptide/peptide^n^	Tripeptide/ peptide	3		x	x	x
157	2.15	253	251	252.11087	252.11101	−0.5	C_12_H_16_O_4_N_2_	6	Phe-Ser or Ser-Phe	Dipeptide	2	[55]	x	x	x
158	2.15	279	277	278.12630	278.12666	−1.3	C_14_H_18_O_4_N_2_	7	Pro-Tyr or Tyr-Pro	Dipeptide	2		x	x	x
159	2.19	455	453	454.15532	454.15560	−0.6	C_16_H_30_O_7_N_4_S_2_	4	Peptide^s^	Peptide	3		x	x	-
160	2.20	280	278	279.12166	279.12191	−0.9	C_13_H_17_O_4_N_3_	7	Di/tripeptide^t^	Di-/Tripeptide	3		x	x	x
161	2.20	318	316	317.15861	317.15869	−0.2	C_13_H_23_O_6_N_3_	4	Tripeptide/peptide^n^	Tripeptide/ peptide	3		x	x	x
162	2.22	229	227	228.14723	228.14739	−0.7	C_11_H_20_O_3_N_2_	3	Leu-Pro	Dipeptide	1		x	x	x
163	2.22	345	343	344.13676	344.13722	−1.3	C_18_H_20_O_5_N_2_	10	Tyr-Tyr	Dipeptide	2		x	x	x
164	2.22	348	346	347.16899	347.16925	−0.7	C_14_H_25_O_7_N_3_	4	Tripeptide^o^	Tripeptide	3		x	-	-
165	2.24	223	221	222.10048	222.10044	0.2	C_11_H_14_O_3_N_2_	6	Phe-Gly	Dipeptide	2		x	x	x
166	2.26	277	275	276.11095	276.11101	−0.2	C_14_H_16_O_4_N_2_	8	Peptide type compound^u^	Dipeptide/ cyclodipeptide	3	[83,84]	x	x	x
167	2.27	294	292	293.13713	293.13756	−1.5	C_14_H_19_O_4_N_3_	7	Di/tripeptide^m^	Di-/Tripeptide	3		x	x	x
168	2.27	295	293	294.15724	294.15796	−2.4	C_15_H_22_O_4_N_2_	6	Leu-Tyr	Dipeptide	1		x	x	x
169	2.28	247	245	246.12108	246.12157	−2.0	C_10_H_18_O_5_N_2_	3	Dipeptide^d^	Dipeptide	3		x	x	x
170	2.29	231	229	230.16275	230.16304	−1.2	C_11_H_22_O_3_N_2_	2	Dipeptide^q^	Dipeptide	3		x	x	x
171	2.29	237	235	236.11557	236.11609	−2.2	C_12_H_16_O_3_N_2_	6	Gly-Phe or Phe-Gly methyl ester	Dipeptide derivative	2		x	x	x
172	2.30	267	265	266.12630	266.12666	−1.3	C_13_H_18_O_4_N_2_	6	Dipeptide^l^	Dipeptide	3		x	x	x
173	2.32	229	227	228.14734	228.14739	−0.2	C_11_H_20_O_3_N_2_	3	Ile-Pro or Pro-Ile	Dipeptide	2		x	x	x
174	2.32	557	555	556.15074	556.14283	14.2	C_24_H_28_O_15_	11	Blumeoside A	Plant metabolite	2	[80]	-	x	-
175	2.34	205	203	204.08986	204.08988	−0.1	C_11_H_12_O_2_N_2_	7	Tryptophan	Amino acid	1	[23,24,25,26,55]	x	x	x
176	2.34	227	225	226.09527	226.09536	−0.4	C_10_H_14_O_4_N_2_	5	Deoxythymidine	Nucleoside	2	[85]	x	-	x
177	2.34	263	261	262.13484	262.13512	−1.1	C_11_H_22_O_3_N_2_S	2	Dipeptide^v^	Dipeptide	3		x	x	x
178	2.35	261	259	260.13704	260.13722	−0.7	C_11_H_20_O_5_N_2_	3	Dipeptide^j^	Dipeptide	3		x	x	x
179	2.37	295	293	294.15764	294.15796	−1.1	C_15_H_22_O_4_N_2_	6	Dipeptide^w^	Dipeptide	3		x	x	x
180	2.38	281	279	280.10551	280.10592	−1.5	C_13_H_16_O_5_N_2_	7	Abenquine C or enantiomer	AA quinone	2	[69,70]	x	x	x
181	2.39	277	275	276.11095	276.11101	−0.2	C_14_H_16_O_4_N_2_	8	Peptide type compound^u^	Dipeptide/ cyclodipeptide	3	[83,84]	x	x	x
182	2.39	295	293	294.12163	294.12157	0.2	C_14_H_18_O_5_N_2_	7	Peptide type compound^p^	Dipeptide or peptide derivative	3	[69,70]	x	x	x
183	2.40	160	158	159.08949	159.08954	−0.3	C_7_H_13_O_3_N	2	Acetylvaline	Amino acid derivative	2	[26]	x	x	-
184	2.41	276	274	275.12709	275.12699	0.4	C_14_H_17_O_3_N_3_	8	Ala-Trp	Dipeptide	1		x	x	x
185	2.45	261	259	260.13728	260.13722	0.2	C_11_H_20_O_5_N_2_	3	Dipeptide^j^	Dipeptide	3		x	x	x
186	2.45	263	261	262.13499	262.13512	−0.5	C_11_H_22_O_3_N_2_S	2	Dipeptide^v^	Dipeptide	3		x	x	x
187	2.46	295	293	294.15803	294.15796	0.2	C_15_H_22_O_4_N_2_	6	Dipeptide^w^	Dipeptide	3		x	x	x
188	2.50	281	279	280.10603	280.10592	0.4	C_13_H_16_O_5_N_2_	7	α-Asp-Phe	Dipeptide	1		x	x	x
189	2.54	245	243	244.17876	244.17869	0.3	C_12_H_24_O_3_N_2_	2	Leu-Leu	Dipeptide	1		x	x	x
190	2.54	281	279	280.10582	280.10592	−0.3	C_13_H_16_O_5_N_2_	7	β-Asp-Phe	Dipeptide	1		x	x	-
191	2.55	263	261	262.13402	262.13512	−4.2	C_11_H_22_O_3_N_2_S	2	Dipeptide^v^	Dipeptide	3		x	x	x
192	2.55	265	263	264.14735	264.14739	−0.1	C_14_H_20_O_3_N_2_	6	Val-Phe or Phe-Val	Dipeptide	2		x	x	x
193	2.58	253	251	252.12127	252.12224	−3.8	C_11_H_16_O_3_N_4_	6	His-Pro or Pro-His	Dipeptide	2		x	x	x
194	2.59	382	380	381.15388	381.15360	0.7	C_17_H_23_O_7_N_3_	8	Tripeptide^x^	Tripeptide	3		x	x	-
195	2.60	293	291	292.10606	292.10592	0.5	C_14_H_16_O_5_N_2_	8	Pyr-Tyr or Cyclo(Glu-​Tyr)​	Dipeptide/ cyclo-dipeptide	2	[83]	x	-	-
196	2.61	192	190	191.06101	191.06162	−3.2	C_7_H_13_O_3_NS	2	Acetylmethionine	Amino acid derivative	2	[26,86]	-	x	x
197	2.66	281	279	280.10557	280.10592	−1.2	C_13_H_16_O_5_N_2_	7	Phe-Asp	Dipeptide	2		x	x	x
198	2.66	295	293	294.15748	294.15796	−1.6	C_15_H_22_O_4_N_2_	6	Dipeptide^w^	Dipeptide	3		x	-	x
199	2.69	263	261	262.13505	262.13512	−0.3	C_11_H_22_O_3_N_2_S	2	Dipeptide^v^	Dipeptide	3		x	x	x
200	2.75	276	274	275.12715	275.12699	0.6	C_14_H_17_O_3_N_3_	8	Trp-Ala	Dipeptide	2		x	x	x
201	2.75	433	431	432.12736	432.12678	1.3	C_18_H_24_O_12_	7	Asperulosidic acid or isomer	Plant metabolite	2	[87,88,89,90,91]	-	x	-
202	2.75	518	516	517.23808	517.23840	−0.6	C_21_H_35_O_10_N_5_	7	Peptide^y^	Peptide	3		x	x	-
203	2.77	306	304	305.13740	305.13756	−0.5	C_15_H_19_O_4_N_3_	8	Thr-Trp or Trp-Thr	Dipeptide	2		x	x	x
204	2.78	295	293	294.12150	294.12157	−0.2	C_14_H_18_O_5_N_2_	7	Peptide type compound^p^	Dipeptide or peptide derivative	3	[69,70]	x	x	x
205	2.81	265	263	264.14732	264.14739	−0.3	C_14_H_20_O_3_N_2_	6	Val-Phe or Phe-Val	Dipeptide	2		x	x	x
206	2.91	248	246	247.10475	247.10559	−3.4	C_10_H_17_O_6_N	3	Pentoseproline or Valinopine or Linamarin	Amadori or Opine amino acid or Plant metabolite	2	[92,93,94]	x	x	x
207	2.99	287	285	286.10512	286.10526	−0.5	C_13_H_18_O_7_	5	Orsellinic acid ester	Endophytic fungi metabolite	3	[16,95,96,97]	-	x	-
208	3.01	248	246	247.10585	247.10559	1.1	C_10_H_17_O_6_N	3	Pentoseproline or Valinopine or Linamarin	Amadori or Opine amino acid or Plant metabolite	2	[92,93,94]	x	x	x
209	3.02	528	526	527.29613	527.29552	1.2	C_24_H_41_O_8_N_5_	7	Peptide^z^	Peptide	3		-	x	-
210	3.11	243	241	242.12691	242.12666	1.0	C_11_H_18_O_4_N_2_	4	Dipeptide^#^	Dipeptide	3		x	-	-
211	3.22	174	172	173.10513	173.10519	−0.3	C_8_H_15_O_3_N	2	Acetylleucine or acetylisoleucine	Amino acid derivative	2	[26]	-	x	-
212	3.29	243	241	242.12608	242.12666	−2.4	C_11_H_18_O_4_N_2_	4	Dipeptide^#^	Dipeptide	3		x	-	-
213	3.37	174	172	173.10538	173.10519	1.1	C_8_H_15_O_3_N	2	Acetylleucine or acetylisoleucine	Amino acid derivative	2	[26]	-	x	-
214	3.37	663	661	662.33214	662.33023	2.9	C_35_H_50_O_12_	11	Saponin	Saponin	3	[98,99]	-	-	x
215	3.50	282	280	281.08955	281.08994	−1.4	C_13_H_15_O_6_N	7	Phenylacetyl-glutamine	Amino acid derivative	2		x	x	x
216	3.76	277	275	276.11052	276.11101	−1.8	C_14_H_16_O_4_N_2_	8	Peptide type compound^u^	Dipeptide/ cyclodipeptide	3	[83,84]	x	-	-
217	4.07	517	515	516.27897	516.27685	4.1	C_19_H_36_O_7_N_10_	7	Peptide^¤^	Peptide	3		-	x	-
218	4.23	247	245	246.10060	246.10044	0.7	C_13_H_14_O_3_N_2_	8	Acetyltryptophan	Amino acid derivative	2		x	-	x
219	4.30	397	395	396.21529	396.21212	8.0	C_17_H_28_O_5_N_6_	6	Tripeptide/peptide^§^	Tripeptide/ peptide	3		-	x	-
220	4.95	201	199	200.10441	200.10486	−2.2	C_10_H_16_O_4_	2	Ramulosin derivative	endophytic fungi metabolite	3	[16,100]	x	x	x

a = Dipeptide containing Leu or Ile and Ser or Thr and Val; b = Dipeptide containing Leu or Ile and Asn or Gln and Val; c = Dipeptide containing Leu or Ile and Lys; d = Dipeptide containing Leu or Ile and Asp or Glu and Val; e = Dipeptide containing Leu or Ile and Arg; f = Tripeptide containing Ala, Asp and Gln or Gln, Glu and Gly or Ala, Asn and Glu or a tetrapeptide containing Ala, Ala, Asp and Gly or Ala, Glu Gly and Gly or the methyl ester of acetylated tripeptide containing Asn, Gly and Ser; g = Dipeptide containing Leu or Ile and Gly or Ala and Val; h = Dipeptide containing Leu or Ile and Gln; or a tripeptide containing Ala, Gly and Leu or Ile; or Ala, Ala and Val; or the methyl ester of an acetylated dipeptide containing Gly or Ala and Lys; or the ethyl ester of Ala-Ala-Ala or a tripeptide containing Gly, Gly and Val; or an acetylated dipeptide containing Thr and Val; or the methyl ester of a tripeptide containing Gly, Gly and Leu or Ile; or the methyl ester of a dipeptide containing or Asn and Leu or Ile; or Val and Gln; or the methyl ester of a tripeptide containing Ala, Gly and Val; i = Dipeptide containing Leu or Ile and Thr; j = Dipeptide containing Leu or Ile and Glu; k = Tripeptide containing Arg, Glu and Val; or Arg, Asp and Leu or Ile; or Gln, Gln and Lys or a tetrapeptide containing Ala, Ala, Asp and Lys; or Ala, Gln, Gly and Lys or a pentapeptide containing Ala, Ala, Gly, Gly and Lys; l = Dipeptide containing Phe and Thr or the methyl ester of a dipeptide containing Phe and Ser or Ala and Tyr; or the ethyl ester of a dipeptide containing Gly and Tyr; or the phenyl methyl ester of a dipeptide containing Ala and Ser; m = Dipeptide containing Gln and Phe or a tripeptide containing Ala, Gly and Phe or the methyl ester of a dipeptide containing Asn and Phe or the methyl ester of a tripeptide containing Gly, Gly and Phe or the phenyl methyl ester of a dipeptide containing Gln and Gly or the phenyl methyl ester of a tripeptide containing Ala, Gly and Gly; n = Tripeptide containing Ala, Glu and Val; or Glu, Glu and Leu or Ile; or Pro, Thr and Thr; or Ala, Asp and Leu or Ile; or an acetylated tripeptide containing Gly, Ser and Leu and Ile; or Gly, Thr and Val; or the methyl ester of a tripeptide containing Ala, Asp and Val or Asp, Gly and Leu or Ile; o = Tripeptide containing Asp, Thr and Leu or Ile; Or Glu, Ser and Leu or Ile; or Glu, Thr and Val; or the methyl ester of a tripeptide containing Asp, Thr and Val; or an acetylated tripeptide containing Ser, Thr and Val; p = Dipeptide containing Glu and Phe or Abenquine B1 or Abenquine B2; q = Dipeptide containing Leu or Ile and Val; r = Tripeptide-amide containing Pyr, Glu and Pro; s = Tetrapeptide containing Cys, Met, Thr and Thr or Met, Met, Ser and Ser; t = Dipeptide containing Asn and Phe or a tripeptide containing Gly, Gly and Phe; or a dipeptide-amide containing Asp and Phe; or an acetylated dipeptide-amide containing Gly and Tyr or the phenyl methyl ester of Gly-Gly-Gly; u = Pyr-Phe or Cyclo(3-OH-Pro-Tyr) or Acetylmethoxy-Trp; v = Dipeptide containing Leu or Ile and Met; w = Dipeptide containing Leu or Ile and Tyr; x = Tripeptide containing Ala, Glu and Tyr; or Asp, Phe and Thr; or Glu, Phe and Ser or the methyl ester of a tripeptide containing Asp, Phe and Ser; y = Tetrapeptide containing Gln, Glu, Glu and Leu or Ile; or a pentapeptide containing Ala, Asp, Asp, Val and Val; or Asp, Pro, Ser, Ser or Leu or Ile; or Ala, Ala, Glu, Glu And Val; or Ala, Ala, Asp, Glu and Leu or Ile; or Ala, Glu, Glu, Gly and Leu or Ile; or Asp, Asp, Gly, Val and Leu or Ile; or Asp, Pro, Thr, Ser, Val; or Glu, Pro, Ser, Ser and Val; or Ala, Glu, Pro, Thr and Thr; z = Pentapeptide containing Pro, Pro, Thr, Thr and Leu or Ile; or Glu, Gly, Pro, Leu or Ile and Leu or Ile; or Ala, Asp, Pro, Leu or Ile and Leu or Ile; or Ala, Glu, Pro, Val and Leu or Ile; # = Cyclo(Glu-Leu); or Pyr and Leu or Ile; or Pyr-Val methyl ester; ¤ = Tetrapeptide containing Ala, Arg, Arg and Asp; or Arg, Arg, Glu and Gly; or an acetylated tetrapeptide containing Arg, Arg, Gly and Ser; § = Tripeptide containing Gln, His and Leu or Ile; or a tetrapeptide containing Ala, Gly, His and Leu or Ile; or Ala, Ala, His and Val; or an acetylalted tripeptide containing Ala, His and Lys; or the methyl ester of a tripeptide containing Pyr, Arg and Pro; or a pentapeptide-amide containing Ala, Gly, Gly, Pro and Pro; ° = Is detected by the [2M + H]+ ion due to the used m/z range.

## Supplementary Materials

The following are available online. Supplementary Figure S1: Alignment of ITS region of S16 strain (KJ649998) with its best GenBank matches *Coniochaeta mutabilis* (DQ93680) and *Humicolopsis cephalosporioides* (KC128659). Supplementary Figure S2: Alignment of ITS region of A (KM068384) and R (KJ649992) strains with their best GenBank matches *Acephala applanata* (AY078147) and *Phialocephala fortinii* (AB671499.2 and AY033087), respectively. Supplementary Table S1: The unidentified metabolites.
