# Correction: Tienaho, J., *et al.* Metabolic Profiling of Water-Soluble Compounds from the Extracts of Dark Septate Endophytic Fungi (DSE) Isolated from Scots Pine (*Pinus sylvestris* L.) Seedlings Using UPLC–Orbitrap–MS. *Molecules* 2019, *24*, 2330

**DOI:** 10.3390/molecules24224017

**Published:** 2019-11-06

**Authors:** Jenni Tienaho, Maarit Karonen, Riina Muilu–Mäkelä, Kristiina Wähälä, Eduardo Leon Denegri, Robert Franzén, Matti Karp, Ville Santala, Tytti Sarjala

**Affiliations:** 1 Faculty of Natural Sciences and Engineering, Tampere University, FI-33101 Tampere, Finland; 2 Natural Resources Institute Finland (Luke), FI-00791 Helsinki, Finland; 3 Natural Chemistry Research Group, Department of Chemistry, University of Turku, FI-20014 Turku, Finland; 4 Department of Chemistry, University of Helsinki, FI-00014 Helsinki, Finland; 5 School of Chemical Engineering, Department of Chemistry and Materials Science, Aalto University, FI-00076 Espoo, Finland

The authors wish to make the following corrections to this paper published in *Molecules*. Two inadvertent errors were found from Table 2 footnotes section:

Footnote k states that “k = Tripeptide containing Arg, Glu and Val; or Arg, Asp and Leu or Ile; or Gln, Gln and Lys or a tetrapeptide containing Ala, Ala, Asp and Lys; or Ala, Gln, Gly and Lys or a pentapeptide containing Ala, Ala, Gly, Gly and Lys”. 

However, it should be “k = Tripeptide containing Arg, Glu and Val; or Arg, Asp and Leu or Ile; or Gln, Gln and Lys or a tetrapeptide containing Ala, Ala, Asn and Lys; or Ala, Gln, Gly and Lys or a pentapeptide containing Ala, Ala, Gly, Gly and Lys”.

Footnote n states that “n = Tripeptide containing Ala, Glu and Val; or Glu, Glu and Leu or Ile; or Pro, Thr and Thr; or Ala, Asp and Leu or Ile; or an acetylated tripeptide containing Gly, Ser and Leu and Ile; or Gly, Thr and Val; or the methyl ester of a tripeptide containing Ala, Asp and Val or Asp, Gly and Leu or Ile”. 

However, it should be “n = Tripeptide containing Ala, Glu and Val; or Glu, Gly and Leu or Ile; or Pro, Thr and Thr; or Ala, Asp and Leu or Ile; or an acetylated tripeptide containing Gly, Ser and Leu or Ile; or Gly, Thr and Val; or the methyl ester of a tripeptide containing Ala, Asp and Val or Asp, Gly and Leu or Ile”.

These changes have no material impact on the conclusions of our paper. We apologize for any inconvenience to our readers.

