# Supplementary material for: Metabolic Profiling of Water-Soluble Compounds from the Extracts of Dark Septate Endophytic Fungi (DSE) Isolated from Scots Pine (Pinus sylvestris L.) Seedlings Using UPLC–Orbitrap–MS
Source: Molecules. 2019 Jun 25;24(12):2330. doi: 10.3390/molecules24122330 (PMC6630819; doi:10.3390/molecules24122330)
Supplement: Supplementary file 1 [file molecules-24-02330-s001.pdf]

Supplementary Materials

Metabolic Profiling of Water-Soluble Compounds from the Extracts of Dark Septate Endophytic Fungi (DSE) Isolated from Scots Pine (*Pinus sylvestris* L.) Seedlings Using UPLC–Orbitrap–MS

Jenni Tienaho [1,2,*], Maarit Karonen [3], Riina Muilu–Mäkelä [2], Kristiina Wähälä [4], Eduardo Leon Denegri [4], Robert Franzén [5], Matti Karp [1], Ville Santala [1] and Tytti Sarjala [2]

[1] Faculty of Natural Sciences and Engineering, Tampere University, FI-33101 Tampere, Finland; karpmatti1@gmail.com (M.K.); ville.santala@tuni.fi (V.S.)

[2] Natural Resources Institute Finland (Luke), FI-00791 Helsinki, Finland; riina.muilu-makela@luke.fi (R.M.-M.); tytti.sarjala@luke.fi (T.S.)

[3] Natural Chemistry Research Group, Department of Chemistry, University of Turku, FI-20014 Turku, Finland; maarit.karonen@utu.fi

[4] Department of Chemistry, University of Helsinki, FI-00014 Helsinki, Finland; kristiina.wahala@helsinki.fi; (K.W.); eleondenegri@outlook.com (E.L.D.)

[5] School of Chemical Engineering, Department of Chemistry and Materials Science, Aalto University, FI-00076 Espoo, Finland; robert.franzen@aalto.fi

* Correspondence: jenni.tienaho@tuni.fi or jenni.tienaho@luke.fi; Tel.: +358-29-532-4986

Table S1. The unidentified metabolites. The intensity of the metabolite in the corresponding fungi extract is shown under the fungal species codes. Intensities over 1×107 are shown in black whereas lower intensities with a grey color. Other ions are also shown if detected. * = Was also detected but not identified by Sun et al. [26]. nd = not detected.

#	rt	$[M + H]^+$	$[M – H]^-$	Exact Mass Measured	A	R	S16	Other Ions
1	0.40	274	272	273.96623*	5×10^7	8×10^6	8×10^6	
2	0.40	388	386	387.94673*	3×10^7	2×10^6	3×10^5	
3	0.40	518	516	517.90331	5×10^6	2×10^5	nd	
4	0.41	218	216	217.97572*	1×10^7	1×10^6	1×10^7	
5	0.46	nd	257	258.07335	6×10^6	1×10^7	3×10^6	
6	0.46	387	385	386.18007	7×10^5	7×10^6	7×10^5	
7	0.47	251	249	250.02328	1×10^7	3×10^7	1×10^7	
8	0.49	308	306	307.21086	6×10^4	2×10^5	7×10^4	
9	0.50	nd	194	195.95317	6×10^6	5×10^6	1×10^7	
10	0.53	219	nd	218.01907	2×10^7	2×10^7	7×10^7	
11	0.53	329	327	328.14845	4×10^7	6×10^7	1×10^7	
12	0.53	nd	371	372.14400	1×10^7	5×10^7	9×10^6	
13	0.53	nd	396	397.13561	2×10^6	2×10^6	8×10^6	
14	0.54	nd	217	218.05522	8×10^8	8×10^8	6×10^8	
15	0.54	221	219	220.03447	2×10^7	2×10^8	2×10^8	
16	0.54	281	279	280.05580	1×10^8	1×10^8	1×10^8	
17	0.55	441	439	440.09453	1×10^8	7×10^7	7×10^6	
18	0.56	353	351	352.14891	4×10^5	7×10^5	9×10^5	
19	0.58	358	nd	357.16752	4×10^8	4×10^8	3×10^8	
20	0.60	nd	194	195.95305	4×10^7	4×10^7	9×10^7	
21	0.61	353	351	352.14790	7×10^5	1×10^6	1×10^6	
22	0.70	nd	238	239.89826	2×10^7	1×10^7	6×10^4	
23	0.70	323	321	322.13713	2×10^5	2×10^6	4×10^5	
24	0.71	265	263	264.11391	9×10^5	2×10^6	4×10^5	
25	0.73	286	284	287.15767	2×10^8	2×10^8	3×10^7	
26	0.74	240	238	239.89876	2×10^7	1×10^7	1×10^5	
27	0.81	409	407	408.17357	1×10^6	4×10^6	1×10^6	
28	0.87	274	272	273.08436	3×10^6	1×10^7	6×10^6	
29	0.96	274	272	273.08439	3×10^6	8×10^6	4×10^6	
30	0.98	nd	522	523.13210	3×10^5	6×10^6	5×10^4	
31	1.05	nd	675	676.20680	9×10^5	nd	nd	
32	1.19	409	407	408.17412	1×10^6	1×10^6	8×10^5	
33	1.21	409	407	408.17412	5×10^5	3×10^6	6×10^5	
34	1.28	588	586	587.10512	nd	nd	9×10^5	
35	1.35	240	238	239.89890	2×10^6	9×10^5	2×10^5	
36	1.74	280	278	279.13032	4×10^6	3×10^6	8×10^6	
37	1.75	714	712	713.21584	1×10^5	nd	nd	
38	1.78	239	237	238.10523	1×10^8	3×10^7	3×10^5	
39	1.78	550	548	549.11226	1×10^7	3×10^7	3×10^7	1099.22937 $[2M + H]^+$; 1097.34277 $[2M – H]^-$
40	1.78	621	619	620.11598	8×10^6	2×10^7	2×10^7	
41	1.79	221	219	220.09434	8×10^6	2×10^6	6×10^5	
42	1.85	501	499	500.02419	3×10^7	5×10^7	6×10^7	
43	1.90	430	nd	429.10509	4×10^5	5×10^6	6×10^5	
44	1.93	219	nd	218.10373	1×10^7	nd	1×10^6	
45	1.98	nd	287	288.09615	1×10^7	2×10^7	8×10^6	
46	1.98	428	426	427.56868	7×10^6	2×10^7	7×10^6	
47	1.99	551	549	550.09609	6×10^7	3×10^8	2×10^8	
48	2.02	430	nd	429.10518	7×10^6	2×10^7	7×10^6	
49	2.04	2630	2628	2629.83072	1×10^5	2×10^5	6×10^5	1315.92264 $[M + 2H]^{2+}$; 1313.90808 $[M – 2H]^{2-}$
50	2.04	nd	2674	2675.83414	3×10^4	9×10^4	2×10^5	1336.90979 $[M – 2H]^{2-}$
51	2.05	636	634	635.11345	1×10^7	3×10^7	9×10^6	
52	2.05	557	555	556.14305	5×10^6	6×10^6	7×10^6	
53	2.06	284	282	283.11683	6×10^6	5×10^6	5×10^7	
54	2.06	430	428	429.10496	3×10^6	1×10^7	3×10^6	
55	2.09	521	519	520.22252	nd	nd	5×10^6	
56	2.12	283	nd	282.07945	1×10^7	3×10^7	1×10^7	565.16650 $[2M + H]^+$; 563.15295 $[2M – H]^-$
57	2.12	398	396	397.13389	8×10^7	7×10^7	4×10^7	
58	2.12	532	530	531.13646	3×10^6	1×10^7	3×10^6	
59	2.15	280	278	279.12977	2×10^7	2×10^7	5×10^6	

60	2.15	379	377	378.10960	nd	7×10^6	nd	
61	2.19	232	nd	231.14696	2×10^8	2×10^8	1×10^9	
62	2.19	429	427	428.06072	3×10^6	3×10^7	2×10^7	857.12567 [2M + H]+; 855.11475 [2M – H]-
63	2.29	532	530	531.13615	7×10^6	2×10^7	9×10^6	
64	2.32	556	554	555.14738	3×10^7	5×10^7	2×10^7	
65	2.34	627	625	626.10191	4×10^7	2×10^8	4×10^7	
66	2.35	572	570	571.14232	1×10^7	1×10^7	1×10^7	1143.28784 [2M + H]+; 1141.27905 [2M – H]-
67	2.38	435	433	434.10237	1×10^6	7×10^5	1×10^6	
68	2.40	458	456	457.57568	1×10^7	2×10^7	1×10^7	
69	2.49	418	416	417.59318	1×10^6	3×10^6	2×10^6	836.18762 [2M + H]+; 834.17896 [2M – H]-
70	2.50	217	215	216.08989	nd	5×10^5	nd	
71	2.61	1149	1147	1148.24490	3×10^5	1×10^5	2×10^6	575.12973 [M + 2H]2+; 573.11517 [M – 2H]2-
72	2.62	860	858	859.19628	1×10^6	1×10^6	2×10^6	
73	2.63	1125	1123	1124.23156	4×10^5	3×10^5	1×10^6	563.12306 [M + 2H]2+; 561.10850 [M –2H]2-
74	2.64	1173	1171	1172.25456	nd	nd	4×10^5	587.13456 [M + 2H]2+; 585.12000 [M –2H]2-
75	2.67	836	834	835.18477	1×10^6	3×10^6	3×10^6	
76	2.74	289	287	288.12139	5×10^5	2×10^7	2×10^5	
77	2.84	430	428	429.59822	3×10^4	5×10^4	nd	
78	2.92	297	295	296.14736	2×10^8	5×10^7	2×10^7	314.18057 [M + NH_3 + H]+
79	2.92	1440	1438	1439.71760	5×10^5	3×10^7	1×10^6	720.86316 [M + 2H]2+; 718.84860 [M–2H]2-
80	2.98	311	309	310.12599	2×10^8	2×10^8	5×10^7	328.15967 [M + NH_3 + H]+
81	3.03	217	215	216.09007	2×10^7	1×10^7	8×10^6	
82	3.03	273	271	272.12620	2×10^6	6×10^6	nd	
83	3.21	341	339	340.17302	4×10^8	2×10^8	3×10^7	358.20670 [M + NH_3 + H]+
84	3.28	355	353	354.15187	4×10^8	4×10^8	7×10^7	372.18558 [M + NH_3 + H]+
85	3.29	nd	815	816.24794	3×10^5	nd	nd	
86	3.30	386	nd	385.12025	1×10^6	5×10^6	3×10^7	771.24628 [2M + H]+; 769.23413 [2M – H]-
87	3.46	385	383	384.19998	3×10^8	1×10^8	3×10^7	402.23282 [M + NH_3 + H]+
88	3.53	544	542	543.19283	6×10^6	2×10^6	9×10^5	
89	3.54	399	397	398.17891	4×10^8	4×10^8	1×10^8	416.21155 [M + NH_3 + H]+
90	3.66	219	217	218.11531	8×10^6	5×10^7	9×10^4	
91	3.69	429	427	428.22640	1×10^8	8×10^7	2×10^7	
92	3.77	443	441	442.20564	2×10^8	2×10^8	1×10^8	460.23773 [M + NH_3 + H]+
93	3.80	406	404	405.19078	6×10^5	nd	nd	
94	3.90	473	471	472.25292	3×10^7	5×10^7	nd	490.28571 [M + NH_3 + H]+
95	4.03	883	881	882.45986	3×10^6	2×10^7	nd	1765.91760 [2M + H]+; 1763.91077 [2M – H]-
96	4.08	1085	1083	1084.04655	nd	7×10^5	nd	
97	4.09	534	nd	533.30479	2×10^7	8×10^7	3×10^7	
98	4.96	395	393	394.19960	nd	3×10^7	nd	

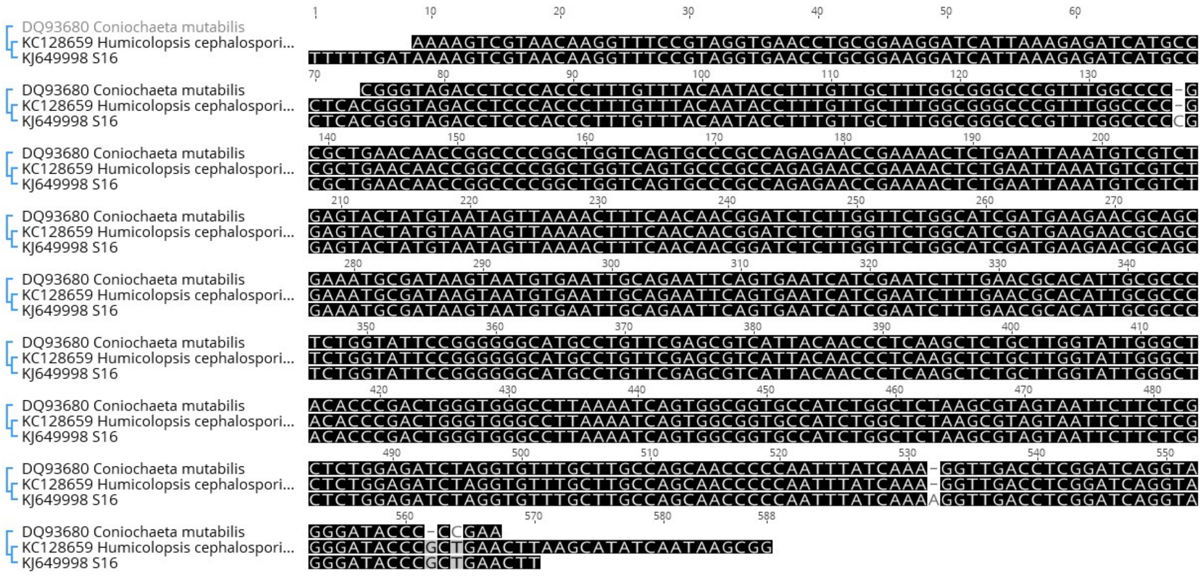

Figure S1. Alignment of ITS region of S16 strain (KJ649998) with its best GenBank matches *Coniochaeta mutabilis* (DQ93680) and *Humicolopsis cephalosporioides* (KC128659).

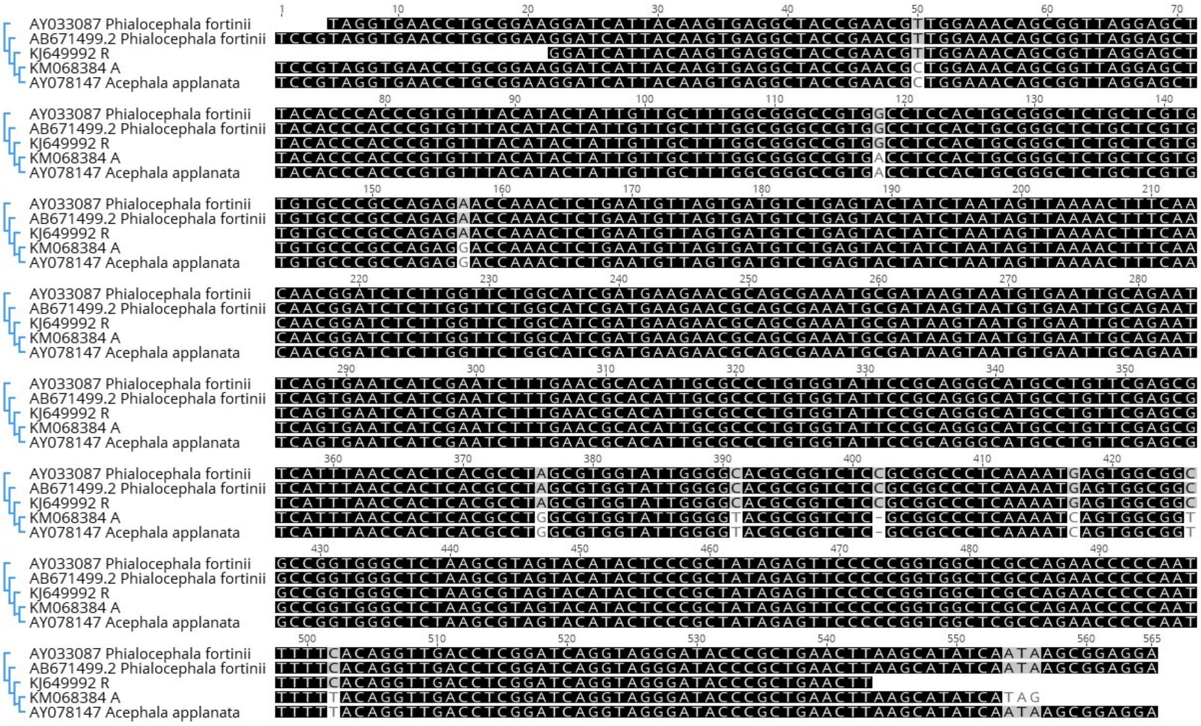

Figure S2. Alignment of ITS region of A (KM068384) and R (KJ649992) strains with their best GenBank matches *Acephala applanata* (AY078147) and *Phialocephala fortinii* (AB671499.2 and AY033087), respectively.
